# FBL promotes hepatocellular carcinoma tumorigenesis and progression by recruiting YY1 to enhance CAD gene expression

**DOI:** 10.1038/s41419-025-07684-z

**Published:** 2025-04-27

**Authors:** Yafei Zhi, Yan Guo, Shiliang Li, Xinyu He, Huifang Wei, Kyle Laster, Qiong Wu, Dengyun Zhao, Jinxin Xie, Shanshan Ruan, Nicholas R. Lemoine, Honglin Li, Zigang Dong, Kangdong Liu

**Affiliations:** 1 State Key Laboratory of Metabolic Dysregulation & Prevention and Treatment of Esophageal Cancer; The School of Basic Medical Sciences, Zhengzhou University, Zhengzhou, China; 2 China-US (Henan) Hormel Cancer Institute, Zhengzhou, China; 3 Tianjian Laboratory of Advanced Biomedical Sciences, Zhengzhou, China; 4 Innovation Center of Basic Research for Metabolic-Associated Fatty Liver Disease, Ministry of Education of China, Zhengzhou, China; 5 Provincial Cooperative Innovation Center for Cancer Chemoprevention, Zhengzhou University, Zhengzhou, China; 6 Cancer Chemistry International Collaboration Laboratory, Zhengzhou, China; 7 Innovation Center for AI and Drug Discovery, East China Normal University, Shanghai, China; 8 Shanghai Key Laboratory of New Drug Design, School of Pharmacy, East China University of Science and Technology, Shanghai, China; 9 Sino-British Research Centre for Molecular Oncology, National Centre for International Research in Cell and Gene Therapy; The School of Basic Medical Sciences, Academy of Medical Sciences, Zhengzhou University, Zhengzhou, China; 10 Center for Cancer Biomarkers & Biotherapeutics, Barts Cancer Institute, Queen Mary University of London, London, UK; 11 Lingang Laboratory, Shanghai, China

**Keywords:** Cancer genomics, Epigenetics

## Abstract

Hepatocellular carcinoma (HCC) is the third leading cause of cancer-related death worldwide. Accumulating evidence suggests that epigenetic dysregulation contributes to the initiation and progression of HCC. We aimed to investigate key epigenetic regulators that contribute to tumorigenesis and progression, providing a theoretical basis for targeted therapy for HCC. We performed a comprehensive epigenetic analysis of differentially expressed genes in LIHC from the TCGA database. We identified fibrillarin (FBL), an rRNA 2′-O-methyltransferase, as an essential contributor to HCC. A series of in vitro and in vivo biological experiments were performed to investigate the potential mechanisms of FBL. FBL knockdown suppressed the proliferation of HCC cells. In vivo studies using cell-derived xenograft (CDX), patient-derived xenograft (PDX), and diethylnitrosamine (DEN)-induced HCC models in *Fbl* liver-specific knockout mice demonstrated the critical role of FBL in HCC carcinogenesis and progression. Mechanistically, FBL regulates the expression of CAD in HCC cells by recruiting YY1 to the CAD promoter region. We also revealed that fludarabine phosphate is a novel inhibitor of FBL and can inhibit HCC growth in vitro and in vivo. The antitumor activity of lenvatinib has been shown to be synergistically enhanced by fludarabine phosphate. Our study highlights the cancer-promoting role of the FBL-YY1-CAD axis in HCC and identifies fludarabine phosphate as a novel inhibitor of FBL.

**Figure:**
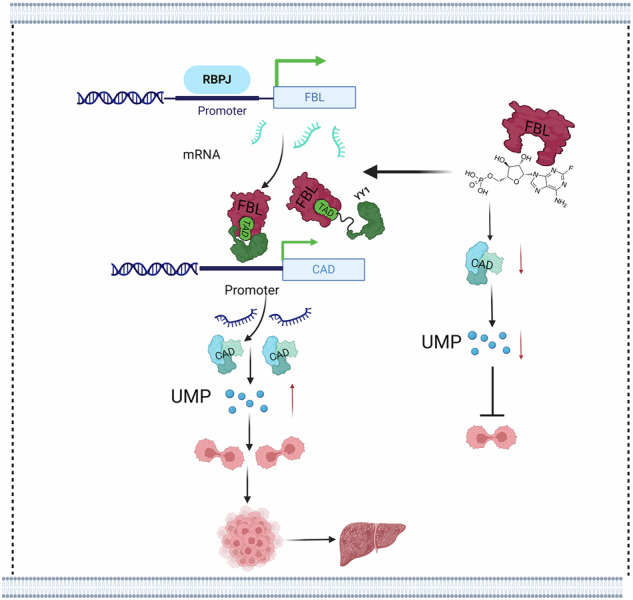
A schematic diagram depicting the FBL-YY1-CAD signaling pathway and its regulatory role in HCC progression.

A schematic diagram depicting the FBL-YY1-CAD signaling pathway and its regulatory role in HCC progression.

## Introduction

Hepatocellular carcinoma (HCC), accounting for nearly 90% of primary liver cancers, remains a highly lethal malignancy [1]. The initial therapeutic option for patients with advanced HCC has been sorafenib, which has remained the standard treatment for over a decade [2, 3]. Nevertheless, the therapeutic outcomes are far from satisfactory, and compared with placebo, sorafenib is associated with a 2.8-month survival in HCC patients [2, 4]. The treatment for advanced HCC has undergone a transformation with the emergence of combination therapies that incorporate immune checkpoint inhibitors [5, 6]. Despite the progress achieved, there remains a high incidence of recurrent HCC that progresses to an incurable and advanced stage. Hence, there is an urgent need to identify novel therapeutic targets in HCC. The most commonly used biomarker for HCC screening is circulating alpha-fetoprotein (AFP), which has unsatisfactory diagnostic efficacy in the screening of early-stage HCC [7]. Therefore, the search for more accurate biomarkers for HCC remains urgent, particularly for early diagnosis and monitoring of treatment outcomes [8, 9].

Accumulating evidence suggests that epigenetic dysregulation contributes to the development and progression of HCC [10]. In recent years, epidrugs have been extensively investigated, and many are currently undergoing clinical trials for hematological malignancies [10, 11]. The first FDA-approved epidrugs include inhibitors of DNMT and HDAC, which are being experimentally tested for tumors such as HCC [12]. However, despite promising results in preclinical studies, therapeutics targeting epigenetic factors have not progressed beyond phase II trials due to concerns regarding patient safety [10]. A detailed investigation of epigenetic alterations will help us identify new targets that can enhance early diagnosis and improve treatment response rates.

FBL, a histone modification transmethylase, can methylate pre-RNA and histone H2A [13, 14]. FBL overexpression contributes to tumorigenesis and is associated with poor survival rates in patients with breast cancer, pancreatic ductal adenocarcinoma cancer, and esophageal squamous cell carcinoma [15–17]. As a downstream target of P53, FBL mediates defects in translation fidelity and IRES-dependent translation initiation, resulting in increased translation of key oncogenes (IGF-1R, C-myc, VEGF-A, and FGF1), ultimately driving tumor initiation and progression [18–21]. A study reports that FBL expression is elevated in tumors and is associated with patient survival in HCC [22]. However, this paper did not investigate the function of FBL in vivo or in vitro, nor did it explore the mechanisms by which FBL promotes the occurrence and development of HCC.

By analyzing all of the differentially expressed epigenetic factors in HCC, we screened the gene with the most pronounced difference in expression, *FBL*. In this study, we identified a significant upregulation of FBL and elucidated its pivotal role in promoting HCC development both in vivo and in vitro. Additionally, our study further revealed that RBPJ positively regulates FBL expression by binding to the FBL promoter. FBL significantly affects YY1 (transcriptional repressor protein YY1) transcriptional activity and regulates the expression of the important gene CAD (multifunctional enzyme carbamoyl-phosphate synthetase II, aspartate transcarboxylase, and dihydroorotase). As a transmethylase, FBL plays a regulatory role in the CAD gene in HCC, not by methylating H2AQ104 but rather through a concealed transactivation domain (TAD) that interacts with YY1. Taken together, these findings suggest that suppressing FBL activity to downregulate CAD may be a promising therapeutic strategy for HCC. Fludarabine phosphate has been extensively used in recent years for the treatment of a variety of hemato-oncologic disorders, lymphomas, acute leukemia, neck cancer, sarcoma, and colon cancer. Here, we identified FBL as a novel target of fludarabine phosphate. Importantly, we demonstrated that the combination of lenvatinib with fludarabine phosphate synergistically enhances its antitumor efficacy.

## Results

### FBL is upregulated in HCC and is associated with poor survival

**Fig. 1:**
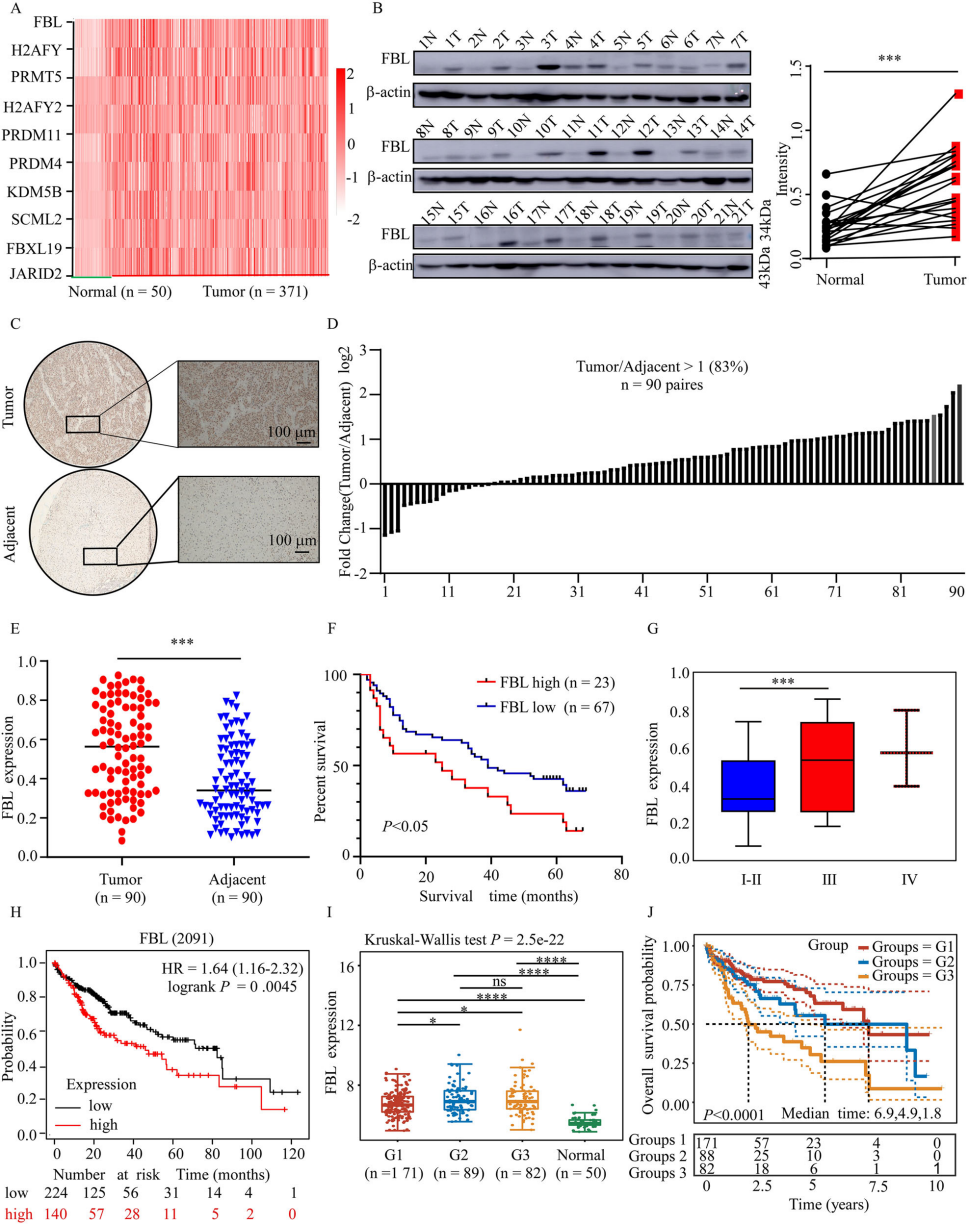
FBL is upregulated in HCC and is associated with poor survival probability. **A** Heatmap displaying the expression of the top ten epigenetic factor genes in the TCGA dataset. **B** Expression levels of FBL in 21 paired human clinical samples with HCC and adjacent tissues. Right panel: normalized FBL protein expression levels relative to actin levels. **C** Representative images of IHC staining of HCC slides using FBL antibody (40× and 100× magnification; scale bar, 100 μm). Statistical analysis was performed for immunohistochemical staining, and FBL expression is indicated as a positive percentage. The tissue microarray in (**C**) from Henan Cancer Hospital comprises 92 paired tumor tissues with clinical information. **D** Graph showing the analysis of the IHC results in paired samples (*n* = 90) in (**C**). A fold change value (tumor/adjacent) >1 indicates FBL upregulation, and a value <1 indicates FBL downregulation in cancer tissues. **E** Graph showing the analysis of the IHC staining results in (**C**). **F** Relationship between the FBL expression level and overall survival in the tissue microarray. **G** FBL expression was examined in patients at various clinical stages via a tissue microarray. **H** Relationships between the FBL expression level and overall survival in patients in the TCGA database, depicted via Kaplan–Meier plotter. **I**, **J** FBL expression in patients with different clinical stages and their survival according to the TCGA database. Data were statistically analyzed via Student’s paired *t*-test in (**B**, **E**, **G**, **I**); Kaplan–Meier analysis in (**F**, **H**, **J**). Error bars represent the means ± SDs. Significance is indicated by *P < 0.05; **P < 0.01; ***P < 0.001.

To investigate the differentially expressed genes (DEGs) associated with epigenetic factors, we analyzed HCC-related data from the TCGA database (https://www.firebroese.org, TCGA-LIHC Cases Project, RNA). DEGs in liver cancer tissues and normal tissues were identified via bioinformatics analysis (Fig. S1A). We subsequently identified 58 differentially expressed epigenetic factors {−log_10_[*P* value > 2] and log_2_[Fold change (Fc) < −1 or > 1]} (Fig. S1B). These 58 DEGs were categorized into three different groups on the basis of their gene function: writers, readers, and erasers (Fig. S1C, D). To validate the gene expression patterns in liver cancer and normal tissues, RT-qPCR assays were conducted (Fig. S1E). Next, we examined the top 10 candidate genes in the TCGA database and revealed that FBL was the most significantly altered gene (Fig. 1A). The mRNA expression of the FBL gene varies across different cancer types (Fig. S1F). Moreover, Western blot analysis revealed a notable increase in FBL protein levels in 81% (*N* = 17/21) of HCC tissues compared with normal tissues (*P* < 0.001; Fig. 1B). Additionally, to assess the prevalence of FBL upregulation in HCC, our investigation was expanded to include 90 paired HCC tumor and adjacent tissues (Fig. 1C). Our findings revealed a significant increase in FBL expression in 83% of primary HCC tissues compared with the corresponding adjacent tissues (Fig. 1D, E). Kaplan–Meier analysis revealed that patients with elevated FBL levels had a lower probability of survival (*P* < 0.05; Fig. 1F). Additionally, clinical correlation analysis revealed an association between FBL expression and clinical stages (Fig. 1G). Similar results were obtained from the clinical relevance analysis of the TCGA data (Fig. 1H–J). These findings strongly suggest that FBL may serve as an oncogene and play a pivotal role in HCC tumorigenesis.

### FBL promotes HCC cell proliferation in vitro and in vivo

**Fig. 2:**
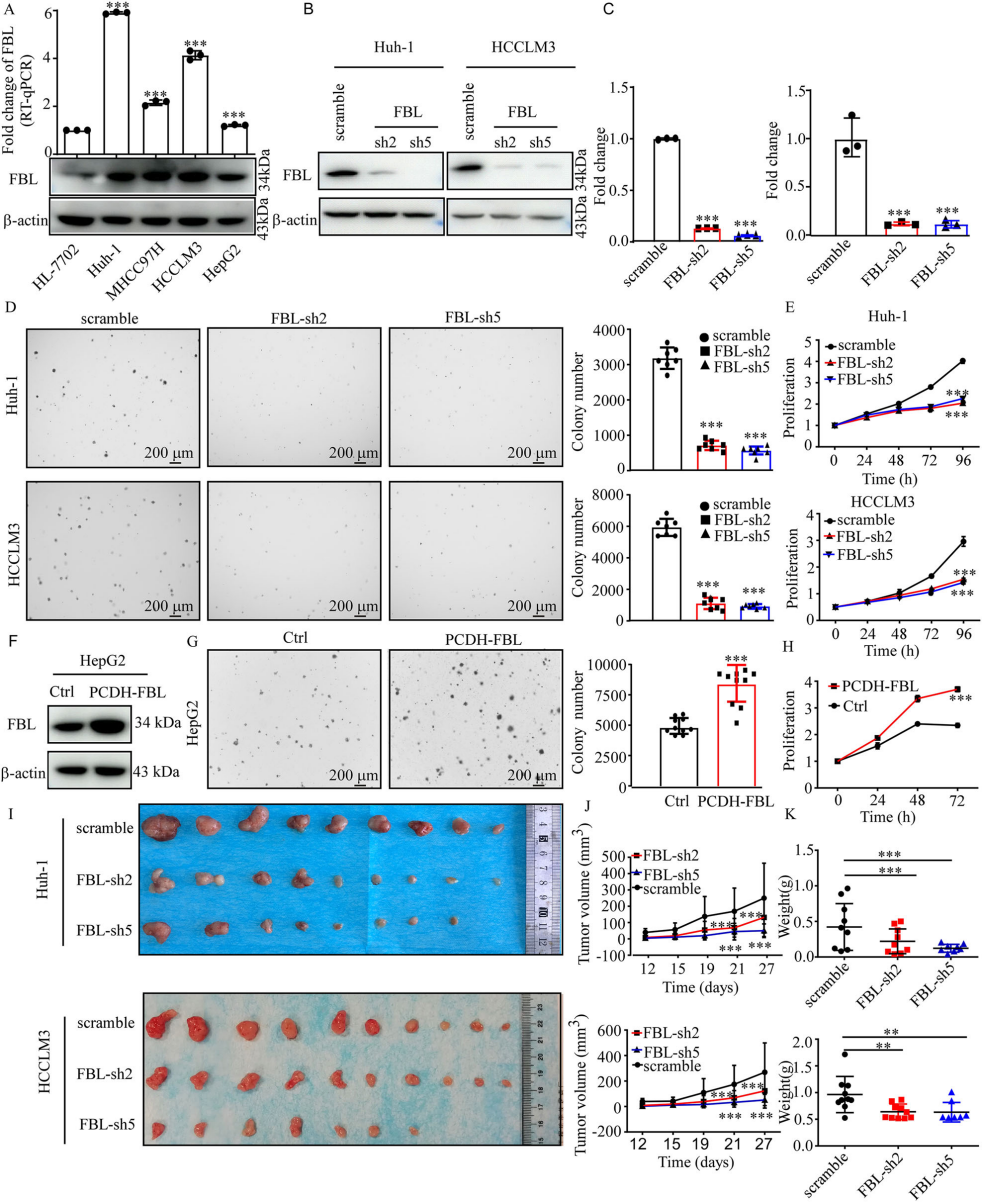
FBL promotes HCC cell growth in vitro and in vivo. **A** Western blot and RT-qPCR were used to evaluate FBL expression in four human HCC cell lines and the normal liver cell line HL-7702. **B**, **F** Establishment of HCC cells with FBL knockdown or overexpression, followed by determination of FBL expression via Western blot. **C** Validation of mRNA expression in FBL-knockdown and scramble cells via RT-qPCR. **D**, **G** Assessment of anchorage-independent growth in HCC cells with FBL knockdown or overexpression. Colonies were quantified via ImageJ-Plus (scale bar: 200 μm). The right panel presents the statistical analysis of colony numbers. **E**, **H** Measurement of cell proliferation through the MTT assay. **I** Huh-1 and HCCLM3 cells stably infected with lentiviral scramble or shFBL were subcutaneously injected into mice; tumors were excised from SCID mice at the end of the experiment. Representative photographs of tumors from each group are provided. **J** Monitoring of tumor sizes every 4–5 days. **K** Measurement of tumor weight after excision; unpaired Student’s *t*-test was employed in (**C**, **D**, **E**, **G**, **H**, **J**, **K**). *P < 0.05, **P < 0.01, ***P < 0.001. The error bars represent the means ± SDs from three independent experiments for in vitro cell experiments.

We subsequently evaluated FBL protein and mRNA levels in the normal human liver cell line (HL-7702) and HCC cell lines. Notably, FBL expression was higher in the HCC cell lines than in the HL-7702 cell line (Fig. 2A). We subsequently constrained our analyses to the Huh-1 and HCCLM3 cell lines owing to their elevated FBL expression levels. To examine the role of FBL in HCC proliferation, we utilized specific shRNAs targeting FBL to establish stable FBL-knockdown cells. Both shFBL #2 and shFBL #5 successfully suppressed FBL expression in the Huh-1 and HCCLM3 cell lines (*P* < 0.0001; Fig. 2B, C). The results from the MTT and soft agar colony formation assays revealed significantly reduced cell growth and colony formation in FBL-knockdown cells (Fig. 2D, E). Conversely, the overexpression of FBL in HepG2 cells led to a 57.7% increase in cell growth and a 71.1% increase in colony formation (Fig. 2F–H). To explore the potential role of FBL in HCC tumor growth in vivo, we subcutaneously injected FBL-knockdown and control cells into nude mice. As expected, the mean tumor weights in the shFBL groups were lower than those in the scramble group (Fig. 2I). Additionally, the tumor volume in the shFBL group was significantly lower than that in the scramble group (*P* < 0.01, Fig. 2J, K). Collectively, these results demonstrate that FBL contributes to the growth of HCC cancer cells both in vitro and in vivo.

### RBPJ regulates FBL expression in HCC

Considering the important role of FBL in HCC, we aimed to elucidate the underlying mechanism for increased FBL expression. Through analysis of the JASPAR, TRAB, and GTRD databases, we identified 23 candidate transcription factors capable of regulating FBL (Fig. S2A). Notably, according to the GEPIA database, RBPJ expression exhibited the strongest correlation with FBL (*R* = 0.59, *P* = 1.78e-14; Fig. S2B, C). To investigate whether RBPJ directly regulates FBL expression, we assessed FBL mRNA and protein levels in RBPJ-knockdown cells. Our findings revealed a significant decrease in both FBL mRNA and protein levels in RBPJ-knockdown HCC cells (Fig. S2D, E). Additionally, luciferase reporter assays revealed reduced FBL promoter activity upon RBPJ suppression (*P* < 0.001; Fig. S2F), supporting the regulatory effect of RBPJ on the FBL promoter. Following the analysis of the FBL promoter region, three potential RBPJ binding sites were identified (Fig. S2G). Subsequent ChIP‒PCR experiments provided further confirmation of the presence of RBPJ on the FBL promoter (Fig. S2H). On the basis of these RBPJ binding sites, we designed 20-base probes containing binding sites modified with biotin (Fig. S2I). A coimmunoprecipitation assay revealed that FBL probes could bind to RBPJ proteins in HCC cells (Fig. S2J). Further investigation via SPR assays using DNA probes based on predicted RBPJ binding motifs revealed that RBPJ could indeed bind to all three DNA probes (Fig. S2K). Collectively, these findings indicate that RBPJ can transcriptionally regulate FBL expression. To evaluate the role of RBPJ in HCC, we knocked down RBPJ in Huh-1 and HCCLM3 cells with specific shRNAs. The knockdown of RBPJ resulted in a significant decrease in both cell growth and colony formation (Fig. S3A, B). Immunohistochemistry (IHC) analysis revealed increased RBPJ protein levels in 90% of tumor tissues compared with adjacent tissues (Fig. S3C–F). TCGA data revealed that RBPJ was highly expressed in tumors and that high RBPJ expression was associated with a lower probability of survival (*P* = 0.027; Fig. S3G, H). Additionally, RBPJ expression was significantly correlated with tumor stage (Fig. S3I). RBPJ regulates gene expression through both NOTCH-dependent and NOTCH-independent pathways, and both pathways can modulate gene expression. To investigate whether FBL is regulated through NOTCH signaling in HCC, we treated cells with the NOTCH inhibitor FI-06 and assessed FBL mRNA and protein expression. The results showed that FBL mRNA expression and protein levels remained unchanged upon FI-06 treatment (Fig. S3J, K). Taken together, these results demonstrate that RBPJ can regulate FBL expression and act as an oncogene in HCC.

### FBL regulates CAD transcription in HCC

**Fig. 3:**
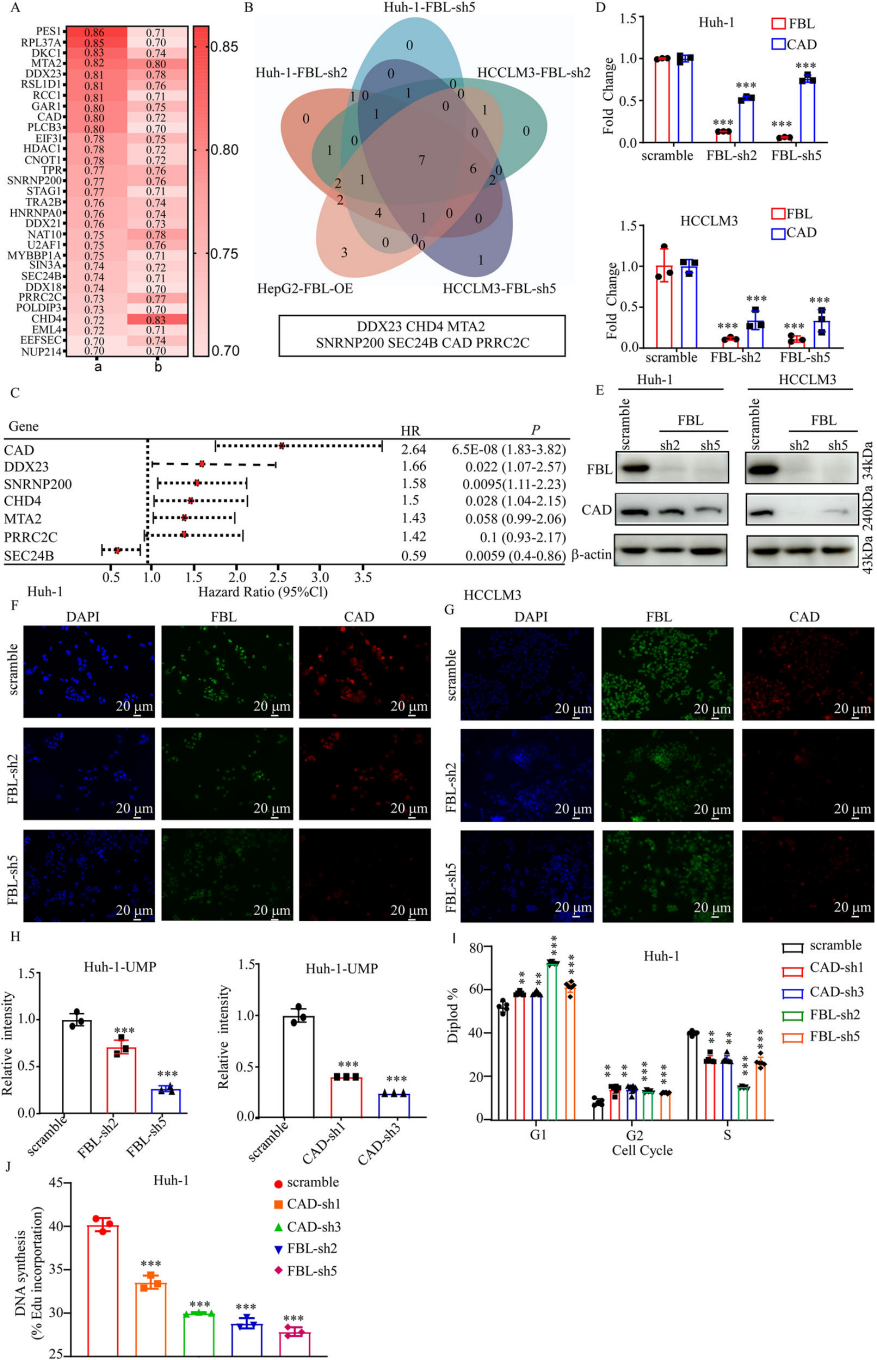
FBL regulates CAD transcription in HCC. **A** Heatmap depicting protein and mRNA expression similarity associated with FBL on the basis of data from Gene Card (a: protein expression similarity; b: mRNA expression similarity). **B** Venn diagram illustrating the overlap of FBL candidate downstream genes. **C** Map illustrating the relationship between the expression levels of candidate genes and overall patient survival. **D** RT-qPCR analysis of CAD expression in Huh-1 and HCCLM3 cells after FBL knockdown. **E** Western blot analysis of CAD protein levels in Huh-1 and HCCLM3 cells after FBL knockdown. **F**, **G** Immunofluorescence assay to assess the expression of FBL and CAD in Huh-1 and HCCLM3 cells after FBL knockdown. **H** UMP LC-MS analysis to determine the relative intensity of UMP in FBL- and CAD-knockdown Huh-1 cells. **I** Cell cycle analysis of FBL- and CAD-knockdown Huh-1 cells. **J** Edu assay analysis of FBL- and CAD-knockdown Huh-1 cells. In all the statistical plots, the data are expressed as the means ± SDs. Significance is indicated by *P < 0.05; **P < 0.01; ***P < 0.001. Student’s *t*-test was used to determine significance in (**D**, **H**, **I**, **J**).

**Fig. 4:**
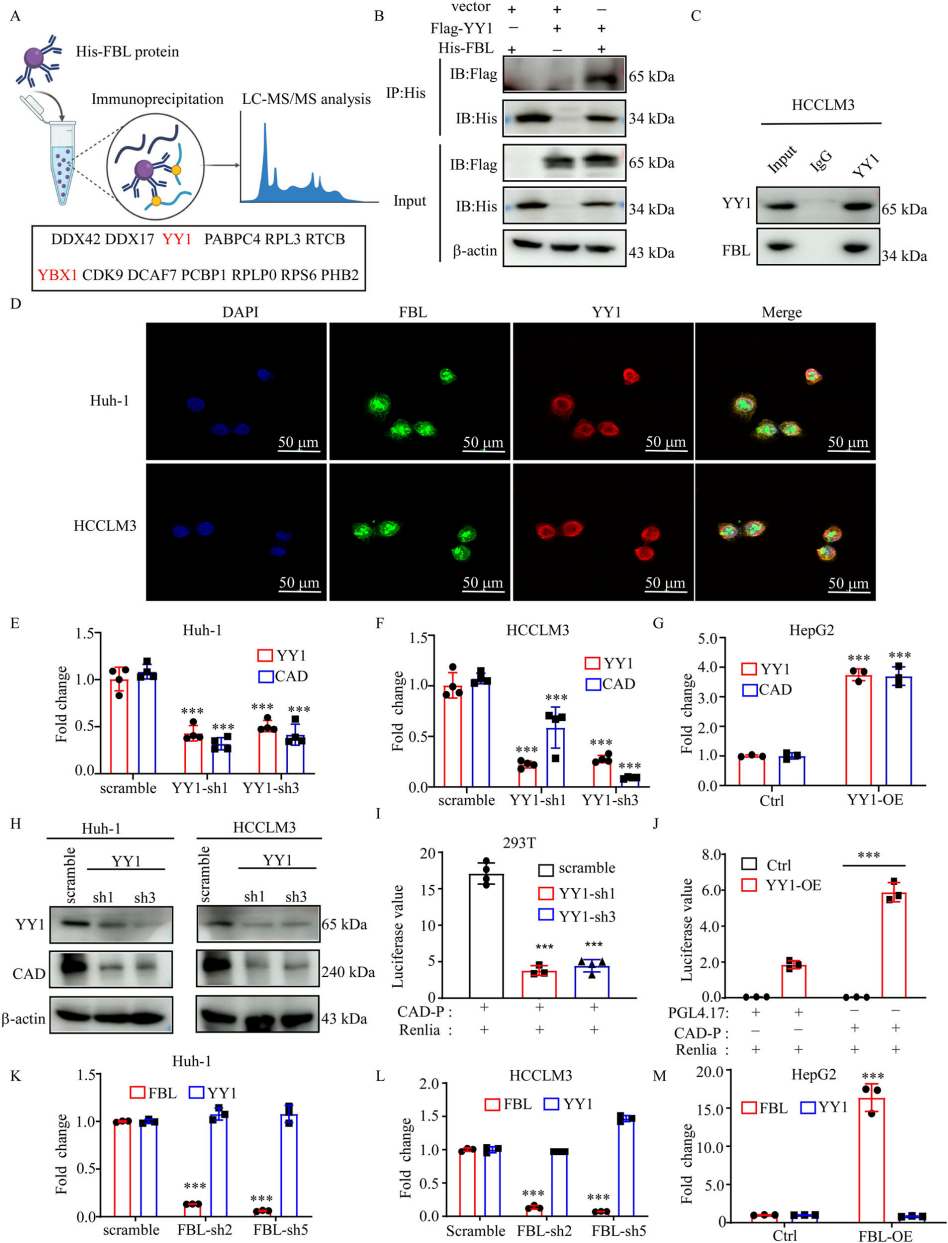
FBL binds YY1 and regulates CAD expression in HCC. **A** MS results of the FBL pull-down assay. **B** In vitro pull-down assay to assess the interactions between FBL and YY1. His-tagged FBL and flag-tagged YY1 plasmids were transiently transfected into HEK293T cells, followed by immunoprecipitation of His-tagged FBL using an anti-His antibody and detection of flag-tagged YY1 via Western blot. **C** Coimmunoprecipitation (co-IP) was used to examine the endogenous interactions between FBL and YY1 in HCCLM3 cells via anti-FBL or anti-YY1 antibodies. **D** Immunofluorescence assay to test the colocalization of FBL and YY1. **E**–**G** RT-qPCR analysis of CAD mRNA expression in HCC cells with YY1 knockdown or overexpression. **H** Western blot to assess CAD expression in HCC cells with YY1 knockdown via two unique shRNAs (#1, #3). **I**, **J** Promoter regions of CAD inserted into the PGL4.17 vector and transfected into YY1-knockdown or YY1-overexpressing HEK293T cells. Luciferase activity was measured at 48 h post-transfection. **K**–**M** RT-qPCR analysis of YY1 mRNA expression in HCC cells with FBL knockdown or overexpression. The error bars represent the means ± SDs. Significance is indicated by ***P < 0.001.

To identify the downstream targets of FBL, we performed an analysis utilizing the gene map database to retrieve a list of genes whose expression patterns were similar to those of FBL. Gene pairs with RNA and protein correlations (*R* ≥ 0.7) were considered (Fig. 3A). Protein similarity was calculated via the Human Integrated Protein Expression Database, and RNA-seq data from GTEx were concurrently analyzed. We performed RT-qPCR to verify the mRNA expression levels of the 31 candidate genes in FBL-knockdown cells and FBL-overexpressing cells (Fig. S4A–C). Seven candidate genes (DDX23, CHD4, MTA2, SNRNP200, SEC24B, CAD, and PRRC2C) were downregulated in FBL-knockdown cells, whereas their expression was upregulated in FBL-overexpressing cells (Fig. 3B). Among these genes, the CAD gene presented the strongest correlation with the survival rate of patients with HCC revealed by the KM plotter database (HR = 2.64, *P* = 6.5E-08; Fig. 3C). Consequently, our attention focused on CAD. Further investigation revealed a significant decrease in CAD mRNA and protein levels in the FBL-knockdown Huh-1 and HCCLM3 cell lines (*P* < 0.001; Fig. 3D, E). Fluorescence intensity analysis revealed a reduction in CAD fluorescence intensity corresponding to decreased FBL fluorescence intensity (Fig. 4F, G). CAD is known to participate in the synthesis of nucleic acid precursors, particularly uridine monophosphate (UMP). As expected, subsequent LC-MS analysis revealed a significant decrease in the UMP concentration in FBL- or CAD-knockdown cells (Fig. 3H). We further examined the impact of FBL and CAD on the cell cycle and DNA synthesis. The results revealed that knockdown of either FBL or CAD induced G1 phase arrest and reduced the DNA synthesis (Fig. 3I, J). In conclusion, our findings suggest that FBL is essential for cell proliferation via CAD-mediated UMP synthesis. To explore the role of CAD in HCC proliferation, we utilized the colony formation and MTT assay. Knockdown of CAD led to a decrease in colony formation in Huh-1 and HCCLM3 cells, as demonstrated by soft agar colony formation assays (Fig. S5A). The MTT assay confirmed the significant inhibition of cell proliferation upon stable knockdown of CAD in Huh-1 and HCCLM3 cells (Fig. S5B). To evaluate the impact of CAD on tumor growth in vivo, we established cell-derived xenografts in nude mice via Huh-1 and HCCLM3 cells depleted of CAD. The data revealed a decrease in tumor growth in the CAD-knockdown cell-derived xenograft mouse model compared with the control (SC vs shRNA1#, *P* < 0.01; SC vs shRNA3#, *P* < 0.001; Fig. S5C–E). These findings led to the conclusion that the repression of CAD inhibits HCC proliferation both in vitro and in vivo. Additionally, we evaluated CAD protein levels in HCC tissues via immunohistochemical (IHC) staining of a tissue array consisting of 92 paired tissues (Fig. S5F). Our IHC results verified that CAD protein levels were increased in 78% of primary HCC tissues compared with the corresponding adjacent tissues (Fig. S5G, H). Kaplan–Meier analysis indicated that HCC patients with high CAD expression had significantly shorter overall survival than those with low CAD expression (*P* = 0.0289; Fig. S5I). Together, our data suggest that CAD plays a crucial role in HCC development.

### FBL binds to YY1 and regulates CAD expression in HCC

To elucidate the mechanism by which FBL regulates CAD expression, we employed a pull-down assay and mass spectrometry to identify 13 potential interacting proteins of FBL (Fig. 4A). Previous studies have demonstrated that the histone transmethylase EZH2 (H3K27me3) binds with the transcription factor c-Myc to mediate gene activation [23]. Given the role of FBL as a histone transmethylase, we sought to investigate whether it interacts with transcription factors to modulate gene expression. Among the candidate proteins, we identified two transcription factors: YY1 and YBX1. The YY1 score (15.85) is higher than YBX1 (10.98). Immunoprecipitation assays confirmed the interaction between FBL and YY1 (FBL does not interact with the YBX1 protein, as verified by pull-down experiments). In the transfected 293T cells, ectopically expressed FBL and YY1 precipitated from each other (Fig. 4B). Furthermore, endogenous YY1 was able to precipitate endogenous FBL in HCCLM3 cells (Fig. 4C). Immunofluorescence assay demonstrated the colocalization of FBL and YY1 in Huh-1 and HCCLM3 cells (Fig. 4D). Silencing of YY1 resulted in a significant decrease in cell viability (Fig. S6A, B). Analysis of YY1 expression in HCC via the TCGA database confirmed elevated YY1 mRNA levels in HCC (Fig. S6C). Patients were stratified into low-risk and high-risk groups on the basis of YY1 expression (Fig. S6D), with the high-risk group exhibiting a worse prognosis (Fig. S6E–G). The areas under the ROC curve (AUCs) were 0.678 at 1 year, 0.614 at 3 years, and 0.587 at 5 years (Fig. S6H). To investigate whether YY1 regulates CAD, we generated stable knockdown cells using specific shRNAs targeting YY1. The results revealed a decrease in CAD mRNA levels in YY1-knockdown Huh-1 and HCCLM3 cells (Fig. 4E, F), whereas the overexpression of YY1 in HepG2 cells resulted in a threefold increase in CAD mRNA levels (Fig. 4G). Additionally, CAD protein levels were significantly decreased in YY1-knockdown cells (Fig. 4H). A luciferase promoter assay revealed that YY1 suppression reduced CAD promoter activity, whereas the overexpression of YY1 increased CAD promoter activity (Fig. 4I, J), indicating that both FBL and YY1 can regulate CAD expression. These results prompted us to explore the relationships among FBL, YY1, and CAD. Interestingly, the mRNA expression level of YY1 did not decrease in FBL-knockdown Huh-1 or HCCLM3 cells (Fig. 4K, L), and the overexpression of FBL did not significantly alter YY1 mRNA expression in HepG2 cells (Fig. 4M). Seven candidate genes (DDX23, CHD4, MTA2, SNRNP200, SEC24B, CAD, and PRRC2C) were identified as FBL downstream genes. In addition to CAD, we used RT-qPCR to test whether other candidate genes are regulated by YY1. As shown in the figure, only CAD was downregulated in YY1-knockdown Huh-1 and HCCLM3 cells (Fig. S7A). Previous studies have reported that YY1 can regulate the expression of the P53, P21, VEGF, VEGFB, AKT, APC, C-MYC, EGFR, COX2, GLUT3, and IL6 genes. In this study, we aimed to investigate whether FBL can also regulate these genes in HCC. The RT‒qPCR results revealed that these genes are not consistently downregulated in FBL-knockdown HCC cells (Fig. S7B). The downstream gene of YY1 may not be regulated by FBL. Previous studies have reported that YY1 and E1A cooperate to increase P53 promoter activity. Additionally, the YY1/BCCIP complex plays a crucial role in regulating the transactivation of P21. Another study revealed that YY1 forms an active complex with HIF-1alpha at VEGF gene promoters, thereby increasing VEGF transcription and expression. These findings suggest that YY1 can cooperate with different proteins to regulate specific target genes. In our study, we found for the first time that FBL binds with YY1 and activates CAD expression, indicating a specific signaling pathway.

### The FBL-TAD domain binds with YY1 to regulate CAD expression

**Fig. 5:**
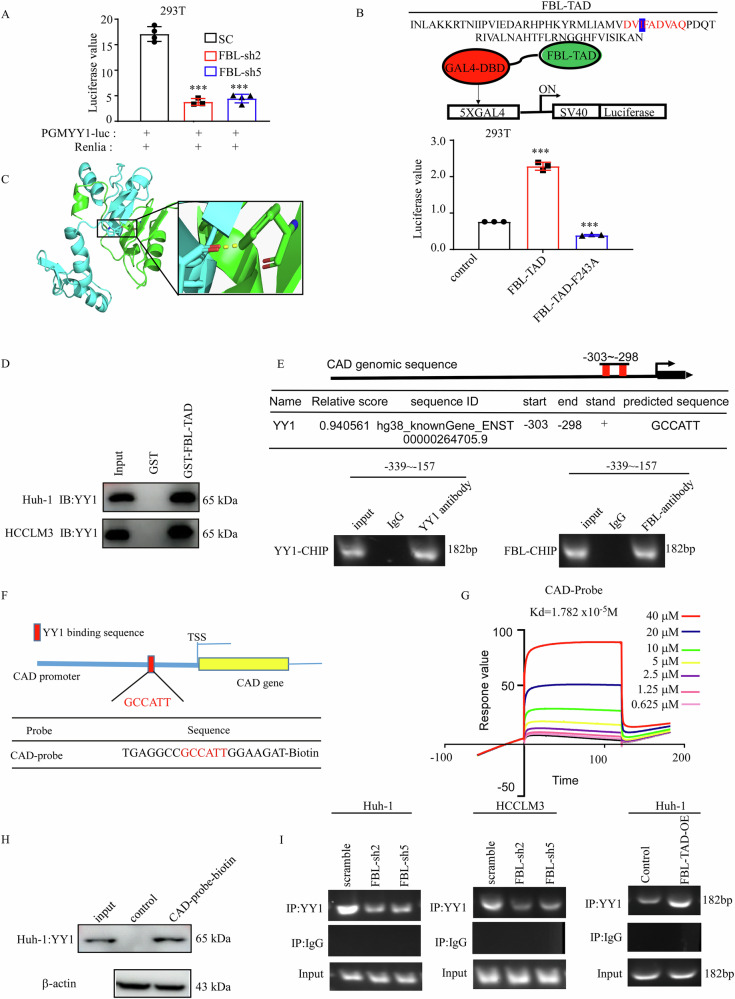
The FBL-TAD domain binds with YY1 to regulate CAD expression. **A** Detection of luciferase activity following the transfection of PGMYY1 plasmids into FBL-knockdown HEK293T cells. **B** Schematic representation of the FBL-TAD domain, highlighted in red, which contains typical Φ-Φ-x-x-Φ motifs. Asterisks denote aromatic residues crucial for transactivation. The GAL4-based luciferase reporter assays show the relative activities of FBL-TAD, both wild type (WT) and mutant, compared with those of the control. The *Y*-axis indicates relative activation normalized to the internal control (Renilla luciferase) and then to the control-transduced mock (*n* = 3; mean ± SD; unpaired two-tailed Student’s *t*-test). DBD represents the DNA-binding domain. **C** Molecular docking assay of the FBL-TAD domain and YY1 protein. Green represents the FBL protein, and blue represents the YY1 protein. **D** Pull-down assay confirming the binding of the FBL-TAD protein and YY1 protein. **E** Prediction of the YY1 binding motif and site in the CAD promoter via JASPAR. ChIP-PCR verified the binding of YY1 and FBL to the CAD promoter. **F** Schematic design of the CAD probes. **G** The ability of the YY1 protein to bind to different concentrations of CAD promoter probes was assessed by SPR. **H** Co-IP experiments were performed to investigate the interactions between the endogenous YY1 protein and CAD probes in Huh-1 cells. The blots have been cropped. **I** ChIP-PCR assay confirming the binding of YY1 to the CAD promoter in FBL-knockdown or FBL-TAD-overexpressing cells. The error bars represent the means ± SDs. In all the statistical plots, the data are expressed as the means ± SDs. Significance is indicated by ***P < 0.001. Student’s *t*-test (**B**) was used to determine significance.

We investigated whether FBL-mediated H2AQ104 methylation is involved in the regulation of CAD expression. First, we found that FBL cannot alter the chromatin state of the CAD promoter, as shown by ATAC sequence analysis (Fig. S8A, C, E). Additionally, we performed CUT & RUN assays using an H2AQ104me antibody and found that the CAD promoter is not occupied by H2AQ104me (Fig. S8B, D, F). To explore the potential regulatory mechanism of FBL on CAD, we constructed a luciferase reporter plasmid containing YY1 binding sites, which are known for their sensitivity in detecting YY1 activation levels. Transfection of this plasmid into FBL-knockdown cells resulted in a significant decrease in YY1 transcriptional activity (Fig. 5A). To elucidate the mechanism underlying FBL-mediated transactivation, we employed 9aaTAD, a transcriptional activity domain prediction tool, to analyze its protein sequence [24]. A putative TAD was identified within amino acids 206-274 of FBL, containing a typical, partially disordered Φ-Φ-x-x-Φ motif (where Φ represents a hydrophobic residue and x represents any residue) (Fig. 5B). Among the luciferase reporters, FBL-TAD exhibited comparable transactivation capabilities. However, the substitution of Phe243 in FBL-TAD with alanine markedly reduced TAD-mediated transactivation (*P* < 0.001; Fig. 5B). Molecular docking assays revealed that the FBL-TAD domain could bind to residues of the YY1 protein (Fig. 5C). Furthermore, FBL-TAD proteins were able to precipitate endogenous YY1 in Huh-1 and HCCLM3 cells (Fig. 5D). Furthermore, examination of the CAD promoter sequence with known transcription factor consensus sequences from JASPAR revealed the presence of a single binding motif (Fig. 5E). ChIP‒PCR confirmed this occupancy (Fig. 5E). On the basis of this YY1 binding site, we designed 20-base probes containing binding sites modified with biotin (Fig. 5F). SPR assays revealed the binding affinity of YY1 for various concentrations of CAD promoter probes (Fig. 5G). Coimmunoprecipitation assays revealed that FBL probes could bind to YY1 proteins in HCC cells (Fig. 5H). We next investigated whether FBL is essential for YY1 binding to the CAD promoter to drive CAD transcription. Through ChIP-PCR analysis, we found that YY1 occupancy at the CAD promoter decreased in FBL-depleted HCC cells. Interestingly, YY1 occupancy increased at the CAD promoter in FBL-TAD-overexpressing cells (Fig. 5I). These findings suggest that the FBL-TAD domain is essential for YY1 binding to the CAD promoter. Collectively, our data suggest that the FBL-TAD domain recruits YY1 to the CAD promoter, thereby activating gene expression, which is independent of FBL-mediated methylation of H2AQ104. FBL primarily functions as an rRNA 2′-O-methyltransferase, responsible for methylating rRNA and H2AQ104. Mutations in the GAR domain, such as the R45A mutation, significantly impact the enzymatic activity of FBL as a ribonuclease [25]. Additionally, the methyltransferase-null mutant, created by replacing D236—A key residue required for FBL to bind its methyl donor cofactor, S-adenosyl methionine (SAM)—effectively abolishes its methyltransferase activity. To investigate the role of FBL in HCC, we transfected these mutant plasmids into HepG2 cells and performed an MTT assay to assess cell proliferation. Interestingly, our results showed that even the enzymatically inactive mutant could still promote cell proliferation (Fig. S8G, H). This finding suggests that FBL may contribute to HCC progression through a non-canonical pathway independent of its methyltransferase activity. We also used ATAC-seq to analyze the chromatin state changes associated with FBL, which affected the gene signaling pathways (Fig. S8I, the gene information is detailed in Supplementary Excel 1). These findings underscore the complex relationships among FBL, YY1, and CAD.

### FBL deficiency attenuates HCC progression in vivo

**Fig. 6:**
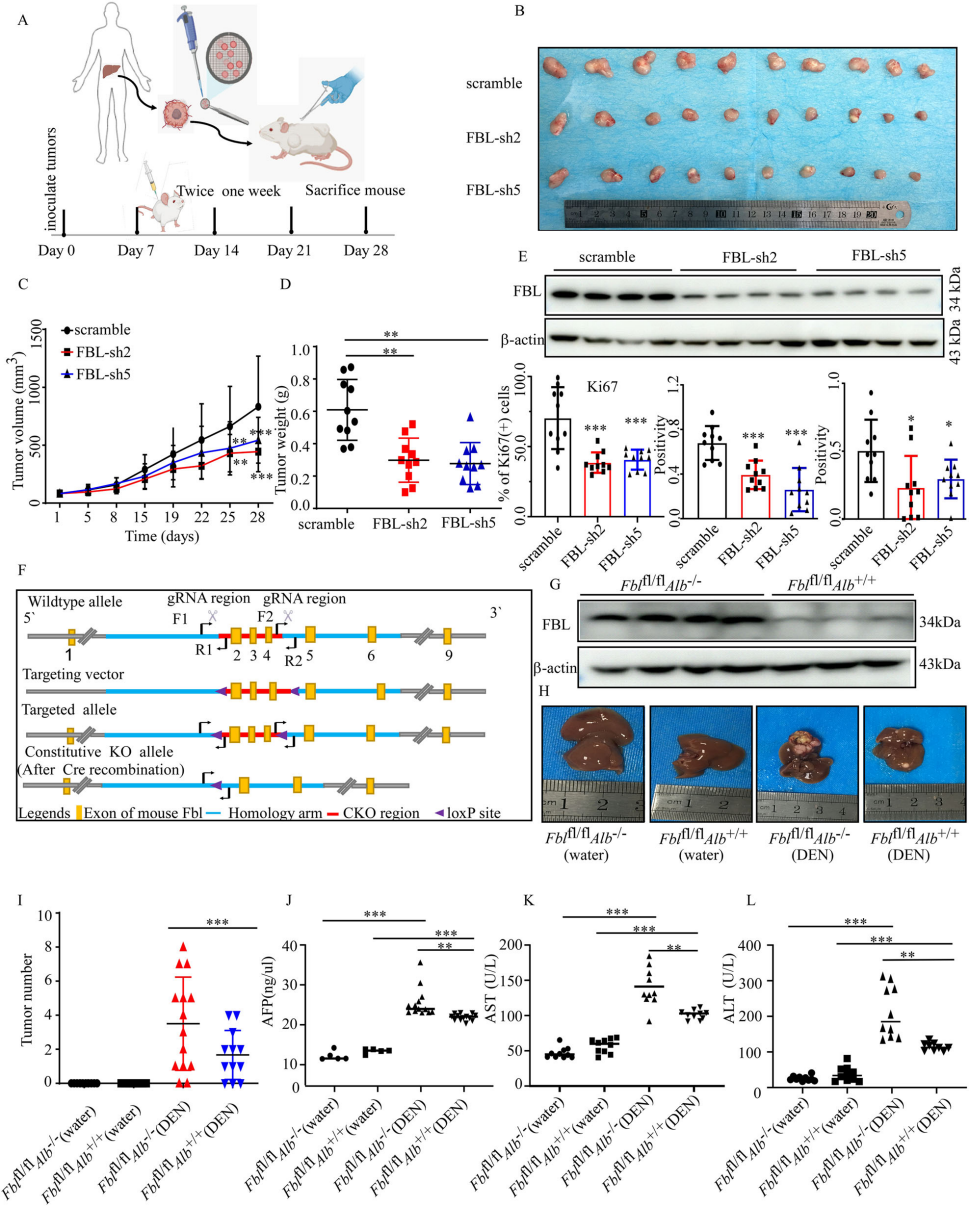
FBL deficiency attenuates HCC progression in vivo. **A**–**E** Tumors derived from the HHG16 model mice were transplanted and then injected with lentiviruses against FBL or a scramble. The tumor volumes were measured, and the tumors were dissected on day 28 after subcutaneous transplantation. **A** Schedule of the FBL PDX model. **B** Photograph of the excised tumor. **C** Growth curves of PDX tumors infected with the indicated lentivirus-mediated shRNAs and scramble. Tumor volume was measured at the indicated time points and statistically analyzed. **D** Graph depicting the weight of PDX tumors at the endpoint. **E** Western blot was used to examine FBL protein expression in different groups of PDX tumors (top). The graph shows the analysis of IHC staining of Ki67, FBL, and CAD from PDX tumors (bottom). **F** Model map illustrating the construction of FBL conditional knockout (CKO) mice by crossing *Fbl* ^fl/fl^ mice with *Alb*-CRE mice. **G** Western blot was used to assess FBL protein expression in the livers of *Fbl*^fl/fl^*Alb*^−/−^ mice and *Fbl*^fl/fl^*Alb*^+/+^ mice. **H** Photograph showing DEN-induced liver damage in different groups. **I** Graph displaying the analysis of tumor number in different groups of mice. **J** ELISA was used to measure the concentrations of AFP in the different groups. **K**, **L** ELISA was used to measure the enzymatic activities of AST and ALT in the different groups. The error bars represent the means ± SDs. Significance is indicated by *P < 0.05; **P < 0.01; ***P < 0.001.

To evaluate the therapeutic potential of targeting FBL in vivo, we generated lentiviruses carrying shRNAs against FBL or scrambled shRNA and delivered them to HCC PDXs to achieve FBL knockdown. Our data revealed that effective FBL depletion in PDXs by lentivirus significantly inhibited tumor growth (Fig. 6A–D), along with a significant decrease in Ki67-positive malignant cells in FBL-knockdown tumors, indicating impaired cell proliferation upon FBL depletion (Figs. S9A, B and 6E). Hematoxylin and eosin (H&E) staining revealed no obvious differences in cell morphology or composition between the scramble and FBL-knockdown groups (Fig. S9A). We observed that FBL protein levels decreased in the FBL depletion groups (Figs. 6E and S9B). Additionally, the results of IHC staining revealed that Ki67, FBL, and CAD decreased after the mice were injected with FBL-knockdown virus (Figs. 6E and S9A). These findings prompted further investigations into the functional importance of FBL in liver tumor development in a mouse model. To better illustrate the effect of FBL on HCC development, we generated HCC FBL liver-specific (CKO) mice by crossing *Fbl*^fl/fl^ mice with *Alb*^+/+^ mice and used DEN to induce HCC [26–28] (Fig. 6F). First, the expression of FBL in mice with different genotypes was determined by Western blot analysis (Figs. 6G and S9C). In addition, *Fbl*^fl/fl^*Alb*^−/−^ mice challenged by DEN developed more tumors compared with *Fbl*^fl/fl^*Alb*^+/+^ mice (Fig. 6H, I). FBL deletion also weakened Ki67 staining in DEN-induced tumors (Fig. S9D). Consistent with the above data, the concentration of AFP was reduced in the *Fbl*^fl/fl^*Alb*^+/+^ mice group following DEN treatment compared to the *Fbl*^fl/fl^*Alb*^−/−^ mice (Fig. 6J). The enzymatic activities of AST and ALT were significantly lower in the *Fbl*^fl/fl^*Alb*^+/+^ group than in the *Fbl*^fl/fl^*Alb*^−/−^ group (Fig. 6K, L). We observed that CAD protein levels decreased in the *Fbl*-CKO mice upon DEN treatment (Fig. S9E). Then, we examined the expression of RBPJ, FBL, and CAD in the DEN-induced HCC mouse model. We found that RBPJ protein expression is significantly upregulated in DEN-induced mice. The expression of its downstream proteins, FBL and CAD, is associated with the upregulation of RBPJ (Fig. S9F). On the basis of these results, we concluded that FBL deficiency inhibits HCC development in vivo.

### Fludarabine phosphate binds with FBL and inhibits HCC progression in vitro and in vivo

**Fig. 7:**
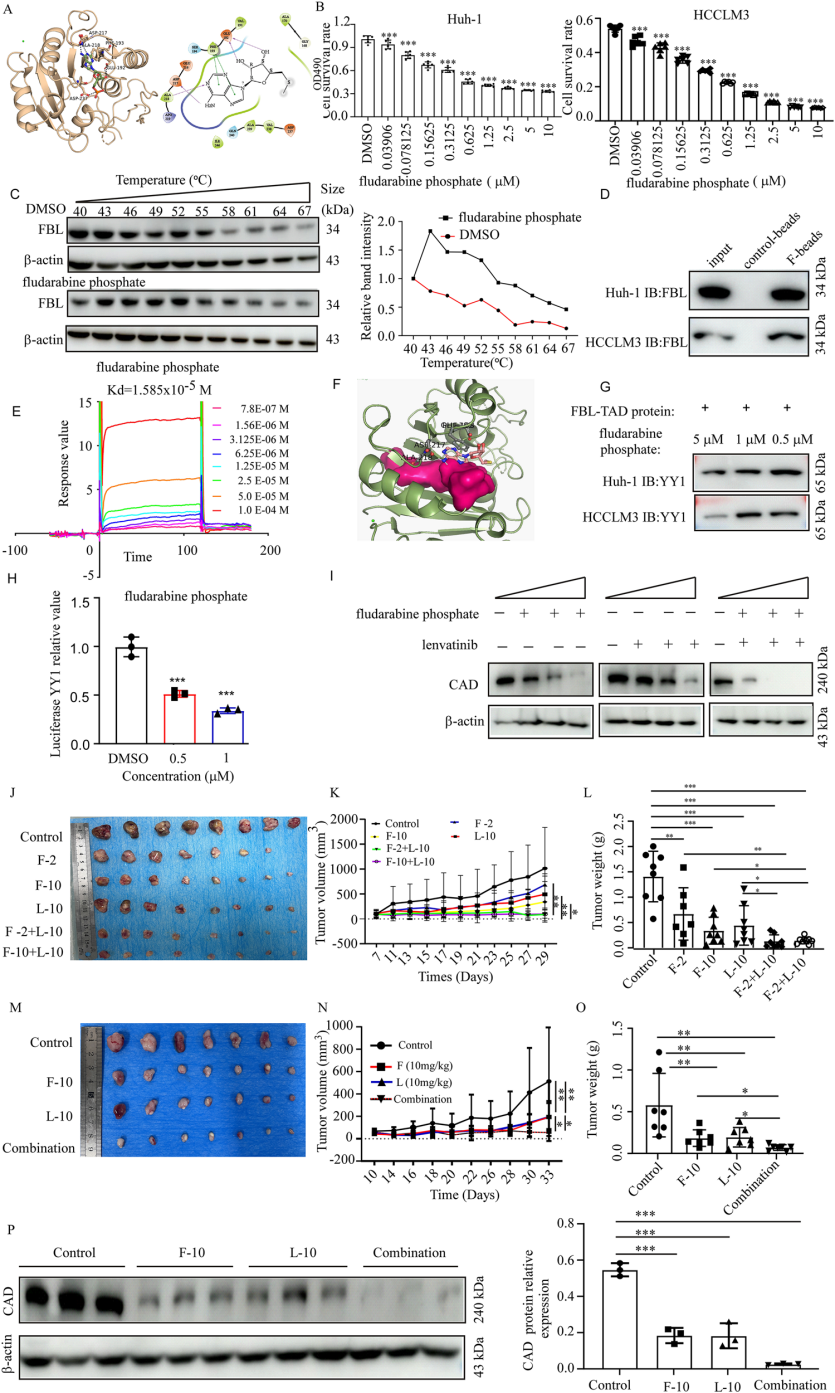
Fludarabine phosphate binds with FBL and inhibits HCC progression in vitro and in vivo. **A** Molecular docking assay demonstrating the interaction between the FBL protein and fludarabine phosphate. **B** MTT assay performed on Huh-1 and HCCLM3 cells treated with different concentrations of fludarabine phosphate. **C** Cellular thermal shift assay (CETSA) assessing the interaction between fludarabine phosphate and FBL. **D** Co-IP assay of FBL protein and fludarabine phosphate-conjugated Sepharose 4B beads, followed by Western blot analysis of the pulled-down proteins. **E** Determination of the binding ability of the FBL protein with different concentrations of fludarabine phosphate via SPR. **F** Molecular docking analysis of the FBL-TAD protein and fludarabine phosphate. **G** IP of FBL-TAD proteins and YY1 in Huh-1 cells treated with different concentrations of fludarabine phosphate. **H** The luciferase activity of YY1 luciferase plasmids transfected into HEK293T cells was measured at 48 h post-transfection after treatment with various concentrations of fludarabine phosphate. **I** Western blot revealing the expression levels of CAD in Huh-1 cells treated with different concentrations of fludarabine phosphate. **J** Female athymic nude mice were injected subcutaneously with Huh-1 cells and treated with fludarabine phosphate (F-2: fludarabine phosphate 2 mg/kg, F-10: fludarabine phosphate 10 mg/kg), lenvatinib (L-10: lenvatinib 10 mg/kg), or a combination (F-2 + L-10: fludarabine phosphate 2 mg/kg + lenvatinib 10 mg/kg, F-10 + L-10: fludarabine phosphate 10 mg/kg + lenvatinib 10 mg/kg). Tumor sizes were monitored every 3 days. Photographs show tumors from CDX mice in different treatment groups. **K** Tumor volumes recorded on the indicated days. **L** Tumor weights measured at the endpoint. **M**–**O** Tumors derived from the HHG44 NOD-SCID model mice were transplanted and treated with different drugs (F-10: fludarabine phosphate 10 mg/kg, L-10: lenvatinib 10 mg/kg, combination: fludarabine phosphate 10 mg/kg + lenvatinib 10 mg/kg). **M** Photographs of excised tumors. **N** Growth curves of PDX tumors treated with different drugs. Tumor volumes were measured at the indicated time points and statistically analyzed. **O** Graph depicting the weight of PDX tumors at the endpoint. **P** Western blot revealing the expression levels of CAD in PDX tumor tissues. Tissue lysates were extracted from PDX tumor tissues across each treatment group, with each blot representing one sample.

As FBL plays a pivotal role in HCC progression, we aimed to identify a potent FBL inhibitor. On the basis of the FBL protein crystal structure, the top 48 chemical molecules were screened from ~1.5 million molecules in the ChemDiv commercial compound library (the 48 molecules are detailed in Table S1). We conducted MTT assays in Huh-1 and HCCLM3 cells to verify their efficacy. Among them, fludarabine phosphate (F47), an FDA-approved drug, exhibited significant effectiveness (Fig. S10A). Using a docking model, we also observed potential binding of fludarabine phosphate to FBL (Fig. 7A). Further investigation of the anti-proliferative effects in Huh-1 and HCCLM3 cell lines revealed that fludarabine phosphate significantly suppressed proliferation and colony formation in a dose and time-dependent manner (Figs. 7B and S10B). We tested the efficiency of fludarabine phosphate after the knockout of FBL via the CRISPR/Cas9 system. As a result, the IC50 of fludarabine phosphate in sgFBL Huh-1 cells (0.657 μM) was greater than that in the Ctrl groups (0.098 μM), and similarly, the IC50 of fludarabine phosphate in sgFBL HCCLM3 cells (0.93 μM) was also greater than that in the ctrl groups (0.08 μM) (Fig. S10C, D). These results indicated that when cells lack FBL, they become less sensitive to fludarabine phosphate. Using a cellular thermal shift assay, we confirmed that fludarabine phosphate binds to FBL (Fig. 7C). Subsequent pull-down assays validated the binding of Sepharose 4B-coupled fludarabine phosphate to the endogenous FBL protein in HCC cells (Fig. 7D). SPR assay further demonstrated the dose-dependent affinity of fludarabine phosphate to FBL (Fig. 7E). Given the influence of the FBL-TAD domain on YY1 transcriptional activity, we conducted protein‒small-molecule docking, revealing the potential binding of fludarabine phosphate to the FBL-TAD domain (Fig. 7F). Moreover, treatment of Huh-1 cells with fludarabine phosphate resulted in competitive binding of fludarabine phosphate to the YY1 protein, as evidenced by Co-IP measurements (Fig. 7G). With respect to the luciferase reporters, we observed decreased transcriptional activity of YY1 in 293T cells treated with different concentrations of fludarabine phosphate (Fig. 7H). RT-qPCR analysis revealed decreased CAD mRNA levels upon treatment with fludarabine phosphate (Fig. S10E), indicating its ability to downregulate CAD through FBL. As FBL functions both as a methyltransferase for rRNA, influencing pre-rRNA synthesis, and as a methyltransferase for H2AQ104 methylation, we used two assays to test whether fludarabine phosphate impairs FBL activity. We used Western blot to assess the H2AQ104 methylation level in cells treated with varying concentrations of fludarabine phosphate. The results indicated that the H2AQ104 methylation level remained unchanged across the different groups (Fig. S10F). To study the impact of fludarabine phosphate on pre-rRNA synthesis, we labeled cells treated with different concentrations of fludarabine phosphate using 5-FUrd. The IF results showed no significant changes across the different groups (Fig. S10G). Combination therapy is gaining increasing attention and has been developed to treat cancer patients by increasing its efficacy, reducing side effects, and overcoming resistance. For liver cancer, the current targeted therapies include sorafenib, lenvatinib, regorafenib, and cabozantinib. We investigated the potential synergistic effects of combining these therapies with fludarabine phosphate. Through MTT assays, we observed that fludarabine phosphate and lenvatinib had combined effects (Fig. S11A). Furthermore, fludarabine phosphate had a synergistic effect with lenvatinib, with a combination index < 1 in HCC cells (Fig. S11B). Our study focused on elucidating the role of the FBL-YY1-CAD axis in promoting HCC progression. Specifically, CAD refers to a multifunctional enzyme complex (carbamoyl-phosphate synthase 2, aspartate transcarbamylase, and dihydroorotase) that plays a crucial role in pyrimidine synthesis. Previous research has suggested that targets of lenvatinib (VEGFR, FGFR, PDGFR, RET, and KIT) regulate pathways involved in cell proliferation, survival, and angiogenesis, thereby indirectly affecting cell cycle progression, DNA synthesis, and pyrimidine synthesis through downstream signaling pathways. We further investigated whether lenvatinib influences CAD expression, and our results revealed that lenvatinib also inhibited the expression of CAD genes (Fig. S11C). We hypothesized that the combined use of these two drugs further inhibits the expression of CAD, thereby exerting a synergistic effect. Our Western blot results demonstrated that the combination of fludarabine phosphate and lenvatinib led to a more pronounced reduction in CAD protein levels than did treatment with either drug alone (Fig. 7I). We utilized a cell-derived xenograft model to examine the antitumor activity of fludarabine phosphate in vivo. The results revealed a significant reduction in tumor weight in the 2 mg/kg (31.7% decrease compared with the control) and 10 mg/kg (75.9% decrease compared with the control) fludarabine phosphate groups, as observed in the Huh-1 CDX model. Additionally, tumor weight decreased when fludarabine phosphate was combined with lenvatinib in the groups receiving 2 mg/kg fludarabine phosphate plus 10 mg/kg lenvatinib (a 90.6% decrease compared with the control group) and 10 mg/kg fludarabine phosphate plus lenvatinib phosphate (89.7% decrease compared with the control group) (Fig. 7J–L). Low-dose fludarabine phosphate and lenvatinib can significantly reduce tumor growth. To evaluate the effectiveness of fludarabine phosphates in a clinical setting, we utilized a PDX model. Our findings indicate that the combination of fludarabine phosphate and lenvatinib resulted in a greater reduction in tumor weight than either drug alone. Specifically, the fludarabine phosphate group presented a 71.1% decrease in tumor weight compared with the control groups, whereas the fludarabine phosphate plus lenvatinib group presented an 85.0% decrease compared with the control groups (Fig. 7M–O). Our Western blot results revealed a more significant decrease in CAD protein levels in the combination treatment group than in the control, fludarabine phosphate, and lenvatinib groups (Fig. 7P). Additionally, the IHC staining results revealed a reduction in Ki67 and CAD positivity levels following drug treatment in the mice (Fig. S11D–I). These findings demonstrate the effectiveness of fludarabine phosphate in treating HCC and its ability to enhance the antitumor effect of lenvatinib.

## Discussion

Liver cancer has a global incidence of ~800,000 cases each year, making it the sixth most common form of cancer and the third leading cause of cancer-related death worldwide. The majority of preclinical studies have shown that epigenetic remodeling can be reversed through drug administration. The range of available epigenetic modifiers and inhibitors is increasing steadily. However, limited clinical trials on HCC patients have revealed notable toxicity. In this study, we focused solely on the FBL gene among 58 genes associated with histone modification identified through the LIHC TCGA database analysis, while other genes were not extensively discussed. Further exploration of additional key factors influencing liver cancer onset and progression is warranted. Additionally, identifying predictive biomarkers for treatment response remains challenging. Epigenetic research holds promise in revealing diagnostic and prognostic biomarkers for HCC and identifying novel therapeutic targets for more effective treatment strategies in the future [10, 29, 30].

Here, we characterized *FBL* as a cancer-promoting gene that is profoundly upregulated and essential for cell proliferation. Additionally, we elucidated that RBPJ is responsible for the upregulation of FBL in HCC and identified CAD, a key enzyme in pyrimidine biosynthesis, as a downstream effector of FBL. Our findings suggest that FBL has the capacity to modulate the transcriptional activity of YY1, a versatile transcription factor that is pivotal for regulating genes involved in diverse biological processes, such as development, cell proliferation, differentiation, DNA repair, and apoptosis [31–35]. Although FBL can influence YY1 transcriptional activity, our findings indicate that FBL alone may not be adequate to fully regulate these targets. Notably, our study focused primarily on the CAD gene. We propose that FBL and YY1 may have additional common downstream molecules, warranting further investigation.

CAD, as a downstream product of FBL and YY1, plays a pivotal role in the synthesis of nucleic acids, active intermediates, and cell membranes[36–40]. The CAD-mediated pyrimidine metabolism pathway is disrupted in a number of cancers (liver, breast, colon, etc.) and is associated with poor clinical outcomes [41]. Lin Guo performed single-cell DNA sequencing on HCC cells from patients and identified CAD as a promising biomarker for the early recurrence of HCC [42]. In this study, we first found that CAD can be regulated by the epigenetic factor FBL. Our initial findings revealed that FBL functions as a chromatin writer and that the methylation of H2AQ104 did not result in chromosome remodeling of the CAD promoter. FBL possesses a concealed TAD that attracts (co)activators and initiates gene expression. Our findings demonstrate that FBL regulates the expression of CAD in HCC cells by recruiting YY1 to the CAD promoter region. While our primary focus was on elucidating the transcriptional coactivation function of FBL, we did not extensively investigate its role as a histone methyltransferase or its subsequent impact on gene regulation. However, we believe that this area merits further exploration.

Fludarabine phosphate is commonly used to treat leukemia and lymphoma. There are a number of studies and clinical trials exploring its potential use in certain solid tumors, including breast cancer, ovarian cancer, melanoma, colorectal cancer, and prostate cancer. Lenvatinib is a new therapeutic agent used for the first-line treatment of unresectable advanced HCC [43–45]. Lenvatinib, an oral multikinase inhibitor, blocks the activation of vascular endothelial growth factor (VEGF) receptors 1 to 3 and fibroblast growth factor (FGF) receptors 1 to 4 [46–49]. In this study, we found that fludarabine phosphate is a new inhibitor of FBL and that its combination with lenvatinib could improve the treatment of liver cancer.

## Methods

### Patient tissues

The clinical tissue samples used in this study were obtained from patients who were diagnosed at Henan Cancer Hospital. All patients provided written consent for the use of tissue samples.

### Cell culture

All cell lines were gained from the Type Culture Collection of the Chinese Academy of Sciences (Shanghai, China) and cultured at 37 °C in 5% CO_2_. Huh-1, HCCLM3, and HepG2 were cultured in DMEM medium containing 10% FBS and 1% penicillin/streptomycin. Cell lines were authenticated by short tandem repeat fingerprinting.

### Animal experiments

NOD/SCID mice (5–6 weeks) were obtained from Vital River (Beijing, China) and housed in a pathogen-free environment designed for immunodeficient mice at 20 ± 2 °C, 50 ± 10% relative humidity, and 12 h light/dark cycles.

### MTT assay and soft agar colony formation assay

An incubation period of 24 h was followed by seeding HCC cells (6 × 10^3^ cells/well) into 96-well plates. The proliferation of cells was measured via the MTT assay after incubation for 0, 24, 48, 72, and 96 h. The cells were pretreated with 5 mg/mL MTT (10 μL/well) and incubated at 37 °C for 2 h. Cells suspended in DMEM and in soft agar were mixed in 6-well plates.

### Immunohistochemistry (IHC) assay and HE staining

Shanghai Outdo Biotech Company (Shanghai, China) provided the HCC tissue microarray, while clinical samples of HCC and adjacent tissues were obtained from Henan Cancer Hospital. Tumor tissues from the PDX mouse model were paraffin-embedded for IHC analysis. Formalin-fixed tumor tissues were paraffin-embedded, sectioned at 4 µm, and placed on microscope slides. IHC experiments were performed with an immunohistochemistry kit (ZSGB-BIO, PV-9001). The paraffin-embedded samples were cut into 4 µm thick sections and stained with H&E (BASO, China). The stained sections were observed under a microscope and photographed. The antibodies used for IHC were anti-FBL (Abcam, AB166630, 1:200), anti-CAD (Proteintech, 16617-AP, 1:200), anti-RBPJ (Proteintech, 14613-AP, 1:100), and anti-Ki67 (Abcam, ab16667.1:100) antibodies.

### Western blot analysis

Protein extracts were prepared via the use of RIPA lysis buffer containing PMSF. Protein samples were incubated at 95 °C for 10 min, subjected to SDS-PAGE, transferred to membranes, and coated with specific primary antibodies. The antibodies used in this study were rabbit anti-fibrillarin (Abcam, AB166630, 1:1000), anti-YY1 (D3D4Q, CST, 1:1000), anti-RBPJ (Proteintech, 16617-AP, 1:200), mouse anti β-actin (Servicebio, ZB15001-HRP, 1:1000), horseradish peroxidase-conjugated anti-rabbit (Santa Cruz, sc-2005, 1:3000) and anti-mouse (Santa Cruz, sc-2005,1:3000) antibodies.

### Dual-luciferase reporter assay

The dual-luciferase reporter assay was performed via a kit from Vazyme Biotech (DL101) following the manufacturer’s protocol. In brief, cells were transfected for 48 h with either the pGL4.17 blank vector or the pGL4.17 vector containing the FBL promoter region, along with the pRL-TK Renilla luciferase plasmid. Afterward, the cells were lysed, and the lysates were analyzed via the Dual-Luciferase Reporter Assay Kit (Vazyme, DL101-01) according to the manufacturer’s instructions.

### Chromatin immunoprecipitation assay

The cells reached 90% confluence in a 10 cm dish. The mixture was subsequently crosslinked in 1% formaldehyde for 10 min at 37 °C, and the reaction was subsequently quenched by the addition of 125 mM glycine in PBS for 2 min at room temperature. The cells were subsequently washed twice with PBS, pelleted, and lysed. Then, the nuclei were lysed, and the extracts were sonicated via an ultrasonic cell disruption system. Chromatin was precipitated via protein A/G magnetic beads (MCE, HY-K0202) in conjunction with the appropriate antibodies. The immunoprecipitated DNA fragments were analyzed via PCR. The following antibodies were used: YY1 (CST, D3D4Q) and Fibrillarin (Abcam, AB166630).

### Transcription factor-binding site analysis

The jet PRIME in vitro DNA transfection reagent (Polyplus) was used to transfect cells with the pGL4.17 reporter vector. Forty-eight hours after transfection, the cells were harvested, and their luciferase activity was assessed via a dual-luciferase assay.

### Cellular thermal shift assay

After incubation with fludarabine phosphate for 2 h, the HCC cells were collected into PCR tubes (100 μL) and incubated at a series of temperatures from 37 °C to 64 °C, with a gradient of 3 °C for 3 min. After being frozen in liquid nitrogen and thawed on ice twice, the supernatant was collected for subsequent Western blot analysis.

### UMP detection

HCC cells were infected with lentivirus containing either scramble shRNA or shRNAs targeting the indicated genes for 24 h, followed by selection with 5 mg/L puromycin for an additional 48 h. The cells were passaged when they reached 90% confluency. Five days post-infection, 5 × 10^6^ cells were collected in 3 replicates for UMP analysis. The cells were resuspended in 800 μL of 80% methanol in water, vigorously vortexed for 2 min, and then stored at −80 °C overnight. The samples were then centrifuged at 12,000 rpm for 20 min at 4 °C. The clear supernatant was collected and filtered through a 0.22 μm filter. Finally, 50 μL of the supernatant was analyzed via an Agilent 6460 Triple Quad LC/MS platform.

### Surface plasmon resonance

Recombinant FBL proteins were coupled onto a CM5 chip (# BR-1005-30) via carboxyl groups on dextran. After incubation, a series of concentrations of fludarabine phosphate flowed through the protein-CM5 system. The binding was tested and analyzed via a Biacore T200 instrument.

### Cell-derived xenograft (CDX) model

The animal experiments in this study were approved by the Ethics Committee of China-US (Henan) Hormel Cancer Institute (Zhengzhou, Henan, China), with approval documents CUHCI2021043. Huh-1 (1 × 10^7^ cells/mouse) and HCCLM3 (1 × 10^7^ cells/mouse) were subcutaneously injected into the right dorsal flank of 5-week-old female athymic nude mice (Beijing Biotechnology Co., Ltd.). The tumor volume was determined at the indicated times via a Vernier caliper and calculated with the following formula: volume = (length) × (width)^2^ × 0.5. Finally, the mice were sacrificed, and the tumors were excised and weighed. During the experimental procedures and outcome assessments, the investigators were blinded to the group allocations. In the experiment, animals were allocated to the experimental and control groups using a random number table. Inclusion and exclusion criteria were pre-established prior to the study. Samples/animals exhibiting [specific condition, e.g., abnormal behavior, failure to meet health standards, or technical errors] were excluded from the analysis.

### Patient-derived xenograft (PDX) mouse model

For the HCC PDX mouse model, tumors were extracted during surgery and implanted into NOD-SCID mice (Vital River Labs, Beijing, China) in a sterile environment. Intraperitoneal (i.p.) injections of fludarabine phosphate at corresponding concentrations in saline or an equivalent volume of saline per injection per day were administered for five consecutive days, followed by 2 days of rest without injections. Lenvatinib-treated mice were gavaged with 10 mg/kg/day DMSO in 100 μL. Tumors were measured every 3 days via a digital caliper, and the tumor volume was calculated via the following formula: volume = (length) × (width)^2^ × 0.5. During the experimental procedures and outcome assessments, the investigators were blinded to the group allocations. In the experiment, animals were allocated to the experimental and control groups using a random number table. Inclusion and exclusion criteria were pre-established prior to the study. Samples/animals exhibiting [specific condition, e.g., abnormal behavior, failure to meet health standards, or technical errors] were excluded from the analysis. For this animal study, the sample size was estimated based on previous literature and practical considerations, even though no formal statistical methods were used for power analysis. The chosen sample size aligns with common practices in similar studies to ensure reliable results.

### Statistical analysis

All the statistical tests were two-sided, and *P* < 0.05 was considered statistically significant. All the statistical analyses were performed via GraphPad Prism 6 and SPSS 22.0 software. In all statistical plots, data were expressed as the mean ± SD. Significance is indicated by ns, *P* > 0.05, *P < 0.05, **P < 0.01, ***P < 0.001. Diversity between subgroups was assessed by student’s *t*-test/Ordinary one-way ANOVA. Consistency between indexes was assessed by Spearman correlation coefficient analysis. Survival proportions were assessed by Log-rank (Mantel–Cox) test.

## Supplementary information

Supplemental Material

Supplementary Excel 1

## Data Availability

The data supporting the findings of this study are not openly available, but are available from the corresponding author upon reasonable request.
