# Supplementary material for: FBL promotes hepatocellular carcinoma tumorigenesis and progression by recruiting YY1 to enhance CAD gene expression
Source: Cell Death Dis. 2025 Apr 27;16(1):348. doi: 10.1038/s41419-025-07684-z (PMC12034760; doi:10.1038/s41419-025-07684-z)

SUPPLEMENTAL MATERIALS
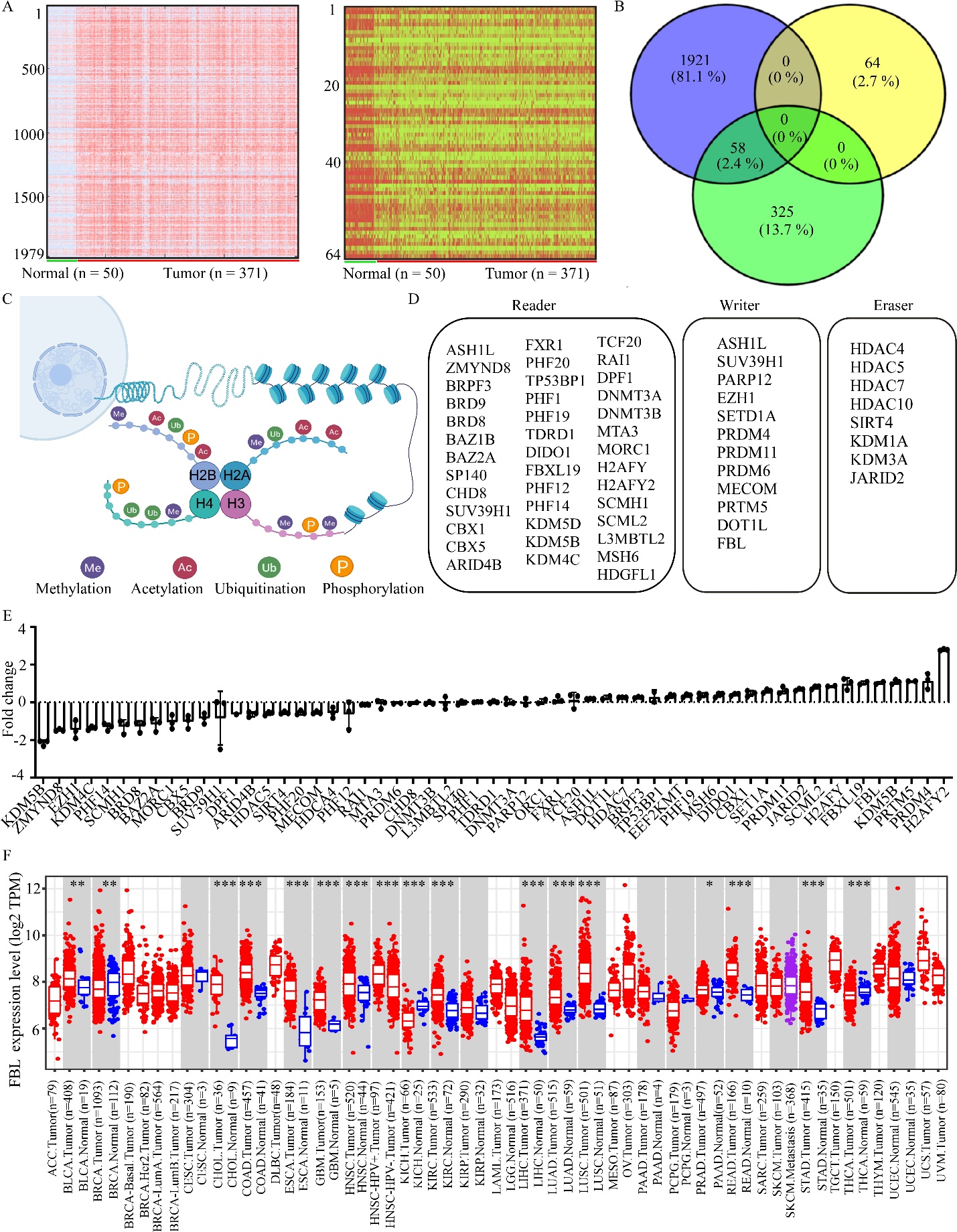


**Supplementary Fig. 1:** **Screen of the histone modification genes in the differentially expressed genes (DEGs) of TCGA database**. A: Heatmap illustrating the expression of positively regulated genes (Left) and negatively regulated genes (Right) in the TCGA dataset. B: Venn diagram illustrating the overlap between histone modification genes and DEGs；Upregulation genes (purple), downregulation genes (yellow)，histone modification genes(green). C: The model map of epigenetic factors modification with histone. D: Classification of 58 histone modification DEGs according to their roles as reader, writer, or eraser. E: mRNA expression levels of the 58 different genes in hepatocellular carcinoma (HCC) tissue samples. F: mRNA expression of the FBL gene across various cancer types. Error bars represent mean ± SD.

**
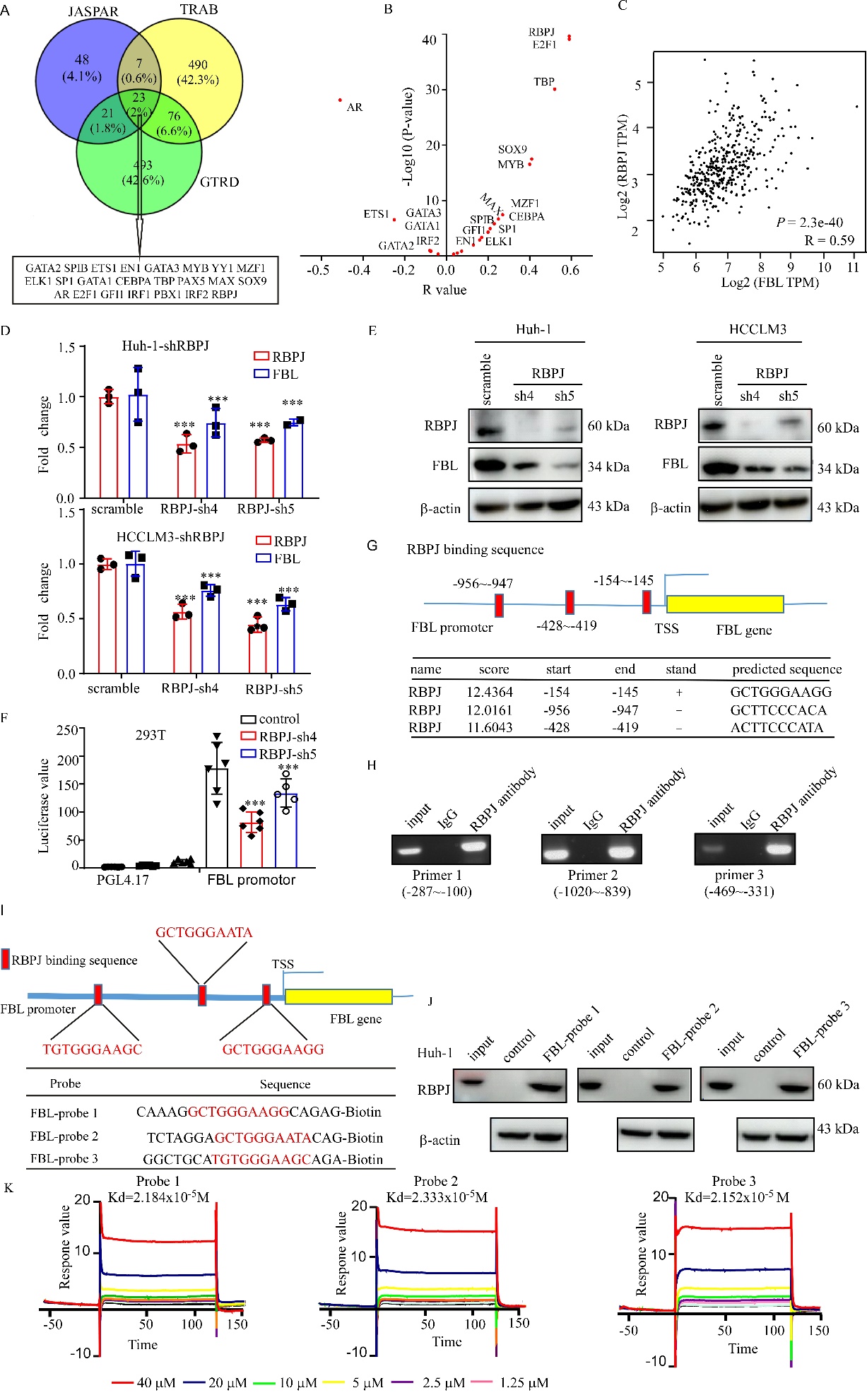
Supplementary Fig. 2: RBPJ regulates FBL expression in HCC.** A: Venn diagram illustrating the overlap of predicted transcription factors regulating FBL expression. B: Heatmap displaying the R and *P* values of 23 candidate transcription factors. C: Correlation analysis between RBPJ and FBL mRNA expression based on data derived from GEPIA. D: RT‒qPCR analysis of FBL mRNA levels in scramble and RBPJ-knockdown cells. E: Western blot analysis of FBL protein levels in scramble and RBPJ-knockdown cells. The blots have been cropped. F: Assessment of relative FBL luciferase promoter activity in 293T cells with RBPJ depletion. Luciferase activity was measured at 48 hours post transfection. G: Prediction of the RBPJ binding site in the FBL promoter via JASPAR. H: CHIP‒PCR validation of RBPJ binding to the FBL promoter region. I: Schematic design of the FBL probes. J: Co-IP experiments investigating the interactions of endogenous RBPJ and FBL probes in Huh-1 cells. The blots have been cropped. K: Evaluation of the binding affinity of the RBPJ protein for different concentrations of FBL promoter probes via SPR. In all the statistical plots, the data are expressed as the means ± SDs. Significance is indicated by ***, *P* < 0.001. Student’s t test was used to determine significance in panels D and F.

**
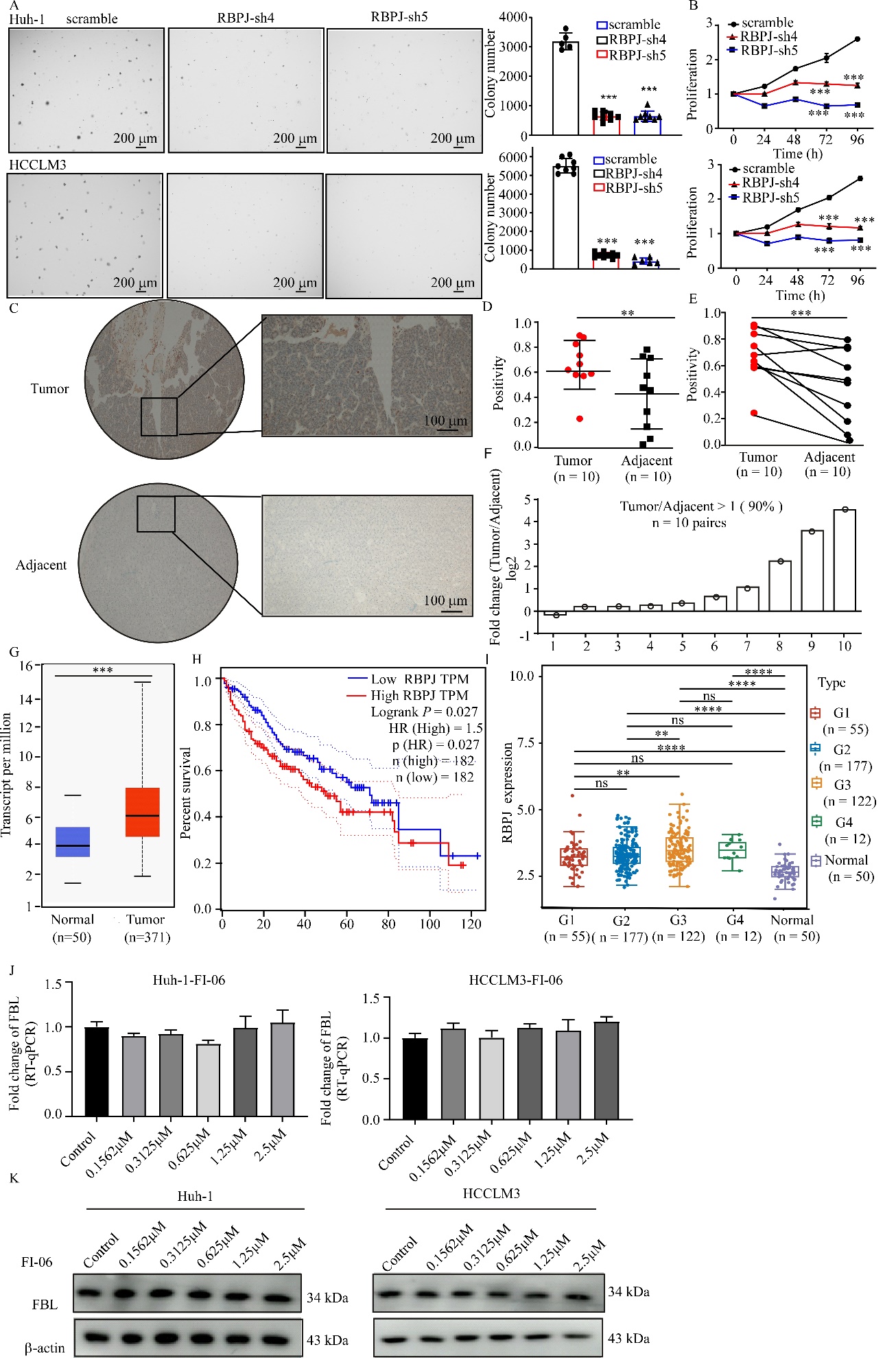
Supplementary Fig. 3: RBPJ function as an oncogene in HCC and predicts poor prognosis in HCC patients.** A: Soft agar colony formation assay measuring the effect of RBPJ knockdown on anchorage-independent cell growth. B: Cell proliferation assessed by MTT assay. C: Representative images of IHC staining of HCC slides using RBPJ antibody (40× and 100× magnification; Scale bar, 100 μm). Statistical analysis performed for immunohistochemical staining, with RBPJ expression denoted as a positive percentage. D, E: Summary of RBPJ expression in paired (D) and unpaired (E) HCC tissue. F: Graph showing the analysis of IHC staining results from tissue array. G: mRNA expression pattern of RBPJ in HCC and normal liver tissues analyzed by UALCAN. H: Relationship between RBPJ expression level and overall survival from the TCGA database. I: Expression of RBPJ in patients with different clinical stages and survival in the TCGA database. J: RT-qPCR analysis to assess FBL mRNA expression in HCC cells treated with NOTCH inhibitor FI-06. K: Western blot analysis to evaluate FBL protein expression in HCC cells treated with FI-06. HCC cells were treated with different concentrations of FI-06 for 48 hours, followed by cell harvesting for RT-qPCR and Western blot analysis. Data statistical analysis performed using Student’s paired *t*-test in A, B, D; Kaplan–Meier analysis in G. Error bars represent mean ± SD. Significance is indicated by *, *P* < 0.05; **, *P* < 0.01; ***, *P* < 0.001; ****, *P* < 0.001.

**
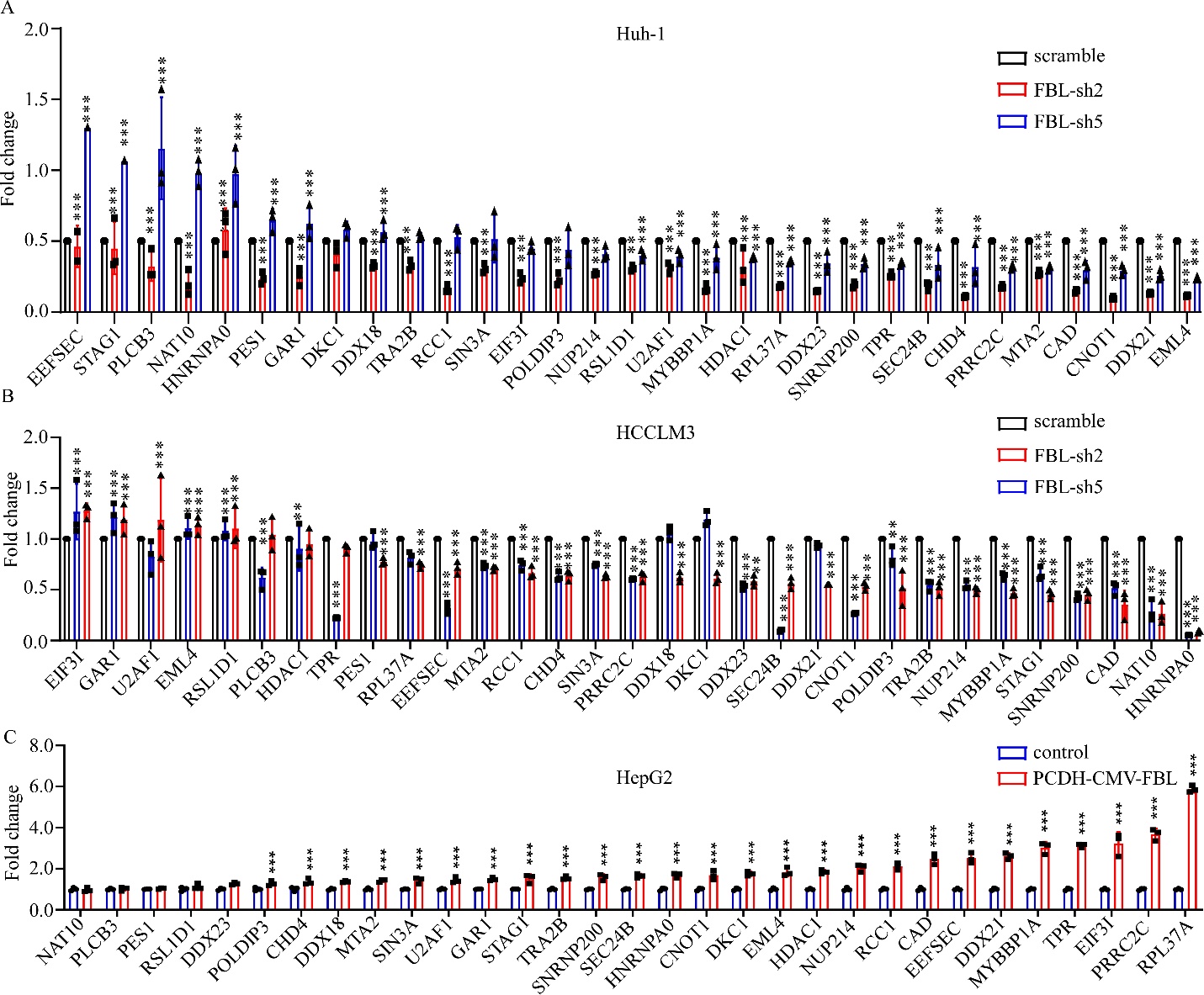
Supplementary Fig. 4: Screen the FBL downstream genes in HCC.** A, B: RT-qPCR analysis of 31 candidate gene mRNA expression in the FBL knockdown Huh-1 and HCCLM3 cells. C: RT-qPCR analysis of 31 candidate gene mRNA expression in the FBL overexpression HepG2 cells. C: RT-qPCR analysis of FBL-correlated genes in FBL knockdown Huh-1 cells. Error bars represent mean ± SD. Significance is indicated by *, *P* < 0.05; **, *P* < 0.01; ***, *P* < 0.001.

**
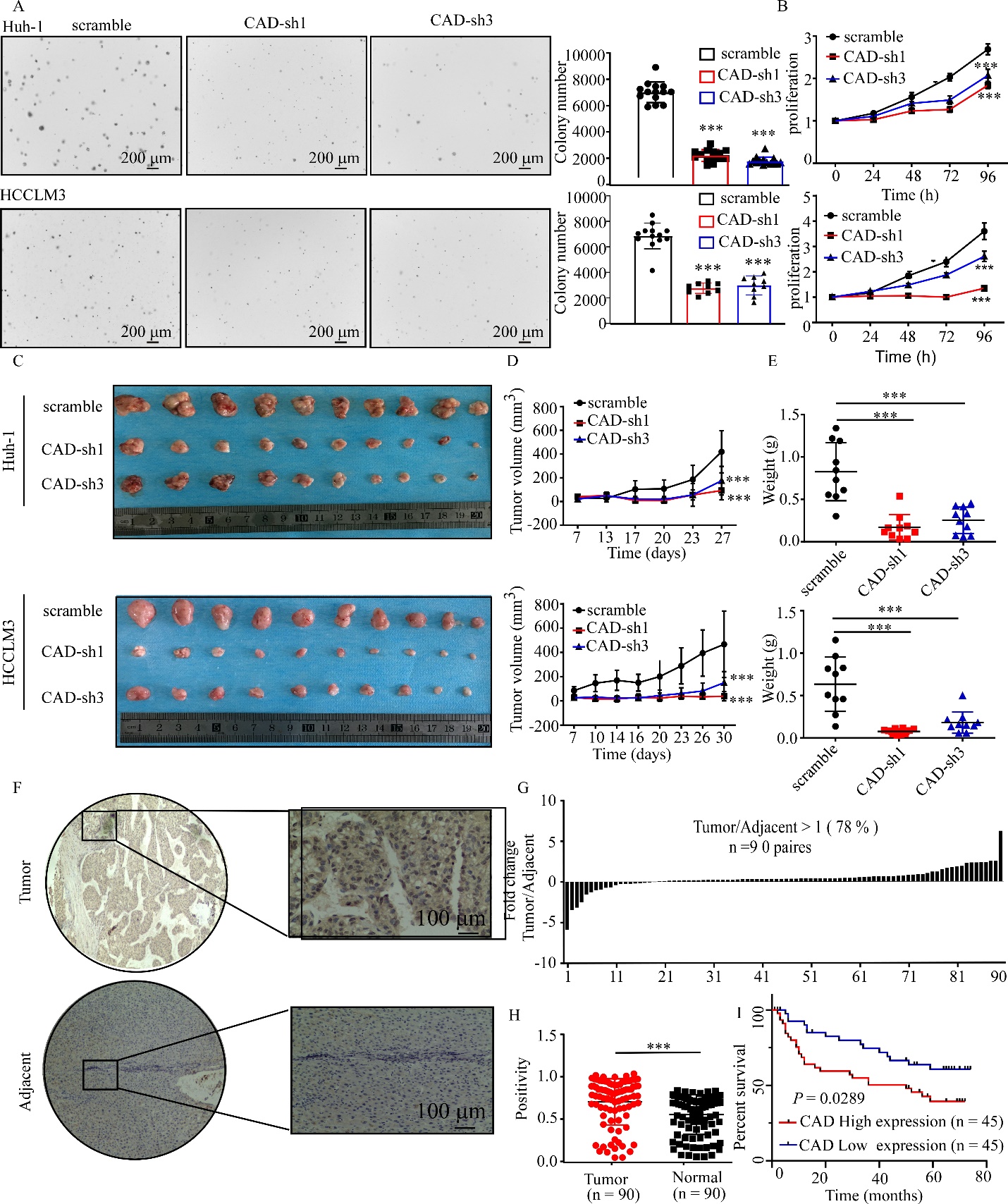
Supplementary Fig. 5: CAD promotes HCC cell growth and is associated with patient survival probability.** A: Soft agar colony formation assay measuring the effect of CAD knockdown on anchorage-independent cell growth. B: Cell viability assessed by MTT assay from day 0 (0h) to day 4 (96h). C: Establishment of a cell-derived xenograft model in nude mice subcutaneously implanted with CAD-knockdown and scramble HCC cells. Photographs of tumors from each group are presented. D: Tumor volumes recorded on the indicated days. E: Comparison of excised tumor sizes through photographs. F: Representative images of Immunohistochemistry (IHC) staining of HCC slides using CAD antibody (40× and 100× magnification; Scale bar, 100 μm). Statistical analysis was performed for immunohistochemical staining, and CAD expression is depicted as a positive percentage. G-H: Summary of CAD expression in paired (G) and unpaired (H) HCC tissues. I: Relationship between CAD expression level and overall survival in tissue microarray. Data were statistically analyzed using Student’s paired *t*-test in A, B, D, E, H; Kaplan-Meier analysis in I. Error bars represent mean ± SD. Significance is indicated by *, *P* < 0.05; **, *P* < 0.01; ***, *P* < 0.001.

**
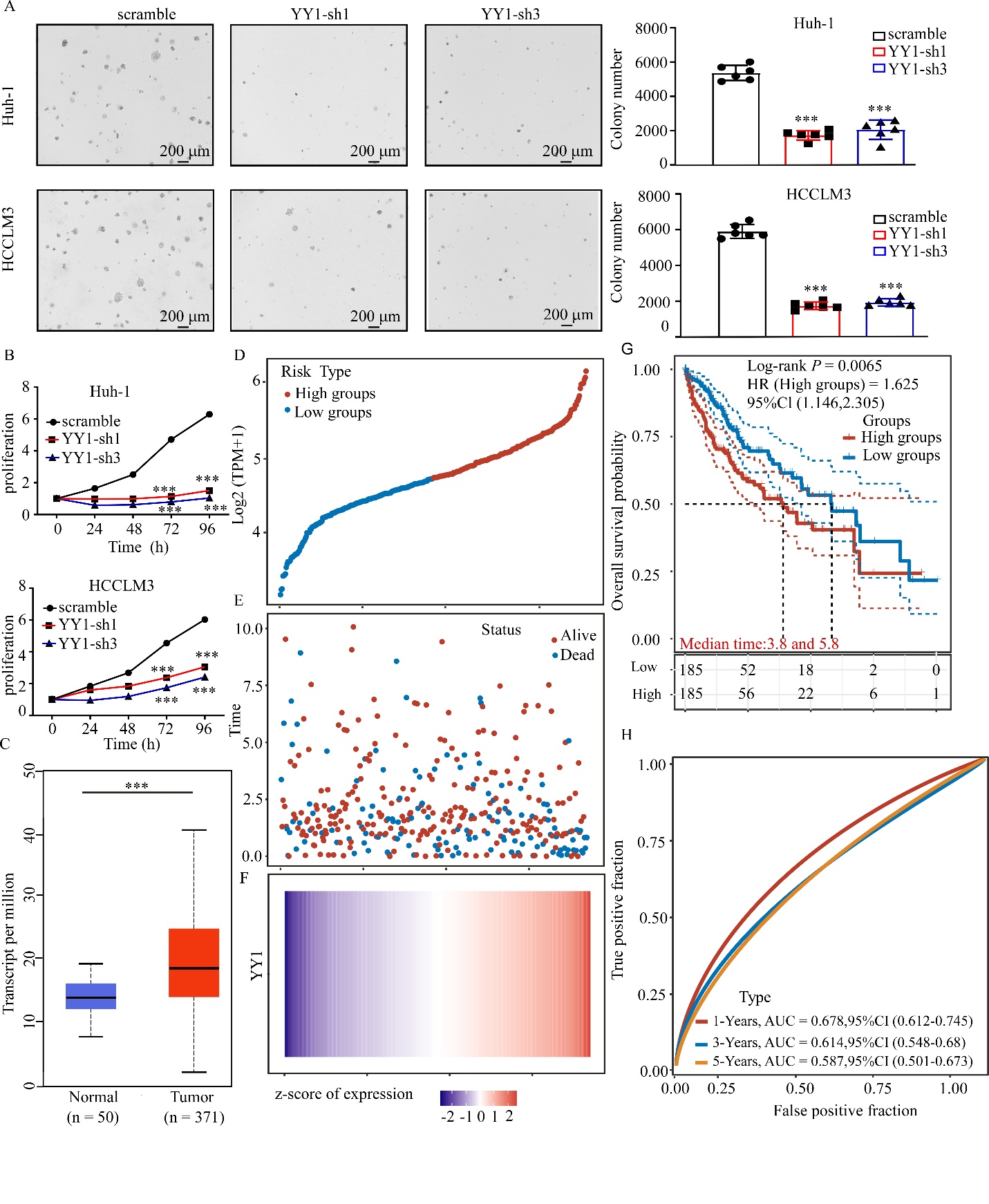
** **Supplementary Fig. 6: YY1 promotes cell growth and is high expression in the HCC.** A: The effect of YY1 knockdown on anchorage-independent cell growth was measured by soft agar colony formation assay. B: Cell viability was determined by MTT assay. C: Graph showing mRNA expression pattern of YY1 in HCC and normal liver tissues analyzed by UALCAN. D: The risk score curve divided the patients into low-risk and high-risk groups. E, F: Patients training group's survival status (E) and heatmap (F) of the expression profiles of the prognostic genes in low-risk and high-risk group. G: Kaplan-Meier survival analysis of the gene signature. H: Time-dependent ROC analysis the of the gene signature. ROC receiver operating characteristic. Bars indicate the mean ± SD from three independent experiments. Asterisks (***, *P* < 0.001) indicate a significant change.

**
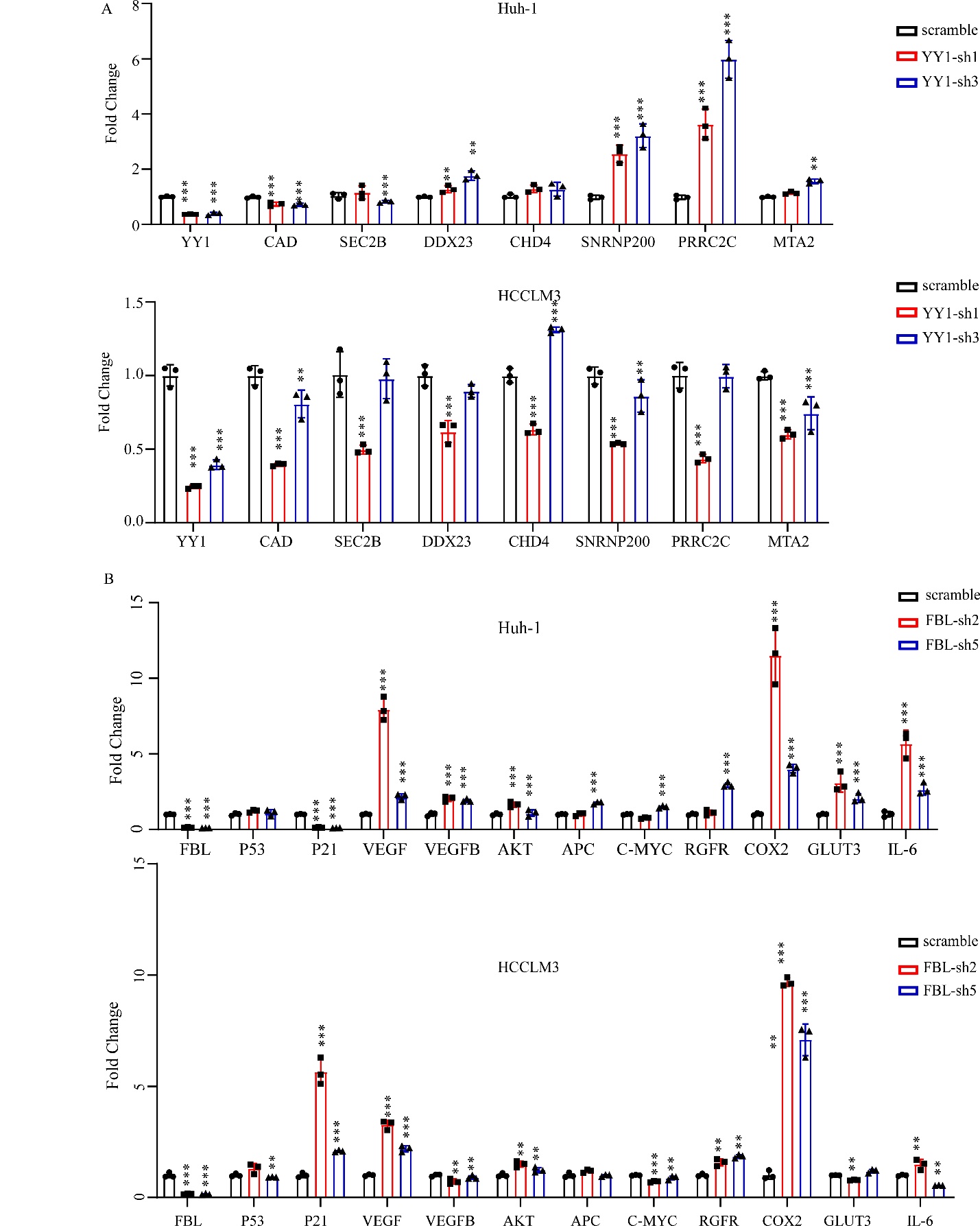
Supplementary Fig. 7: The downstream genes of FBL and YY1.** A: RT-qPCR to test the FBL downstream gene expression in the YY1 knockdown HCC cells. B: RT-qPCR to test the YY1 downstream gene expression in the FBL knockdown HCC cells. Error bars represent mean ± SD. Significance is indicated by *, *P* < 0.05; **, *P* < 0.01; ***, *P* < 0.001.

**
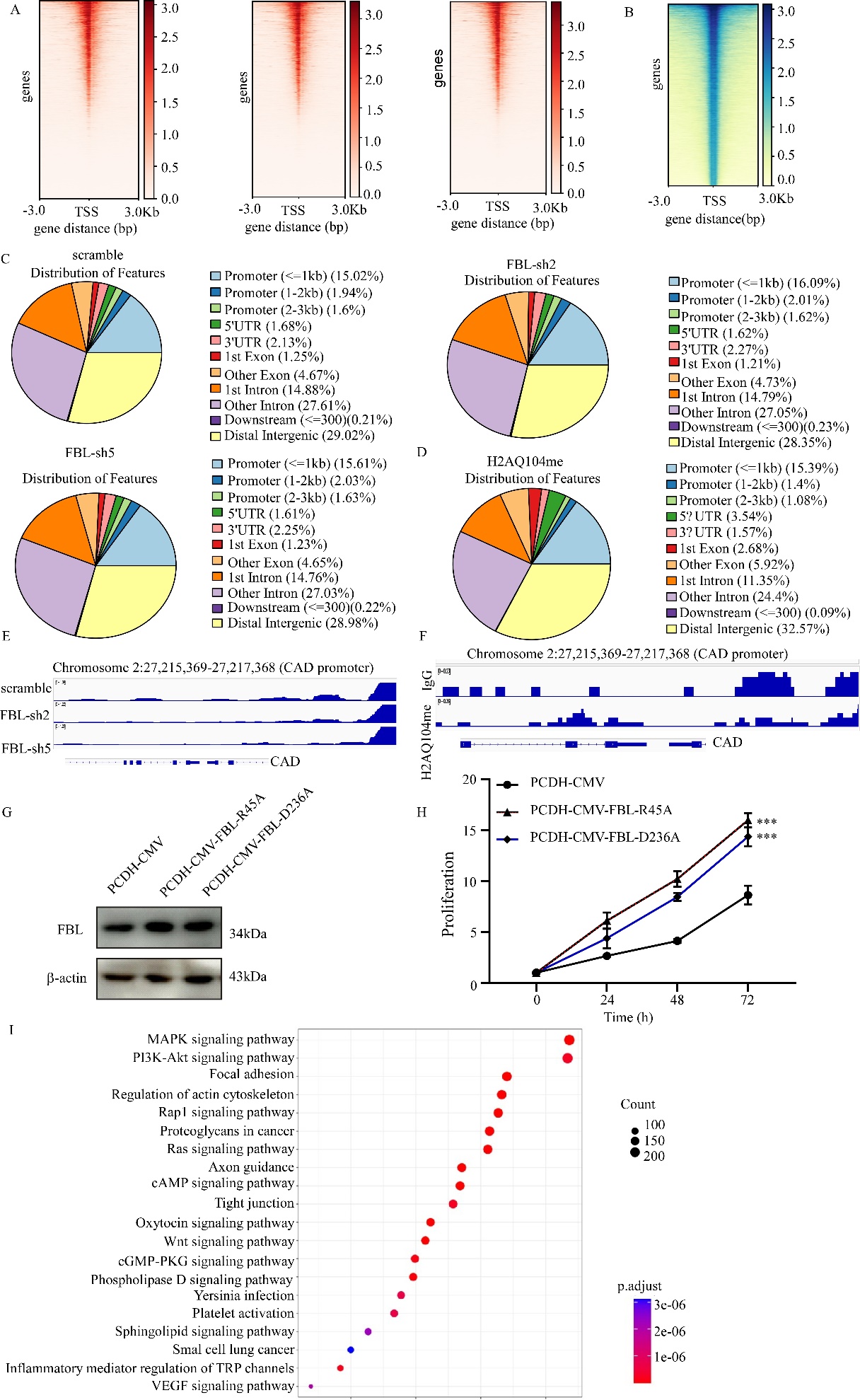
Supplementary Fig. 8: FBL knockdown did not cause the opening of CAD promoter chromatin.** A: Averaged signal intensities and heatmaps for FBL knockdown and scramble cells. B: The enrichment heatmap of transcriptional start site using H2AQ104me antibody in the HCCLM3. C, D: The proportion of peak in the different groups located on the genome. E: IGV views CAD promoter of ATAC result in FBL knockdown and scramble cells. F: The enrichment of H2AQ104me antibody in the CAD promoter compared to IgG. G: Western blot analysis to assess FBL protein expression in the control and mutant groups. H: MTT assay to evaluate cell proliferation in the control and mutant groups. I: KEGG pathway enrichment analysis of genes involved in chromatin opening mediated by FBL.

**
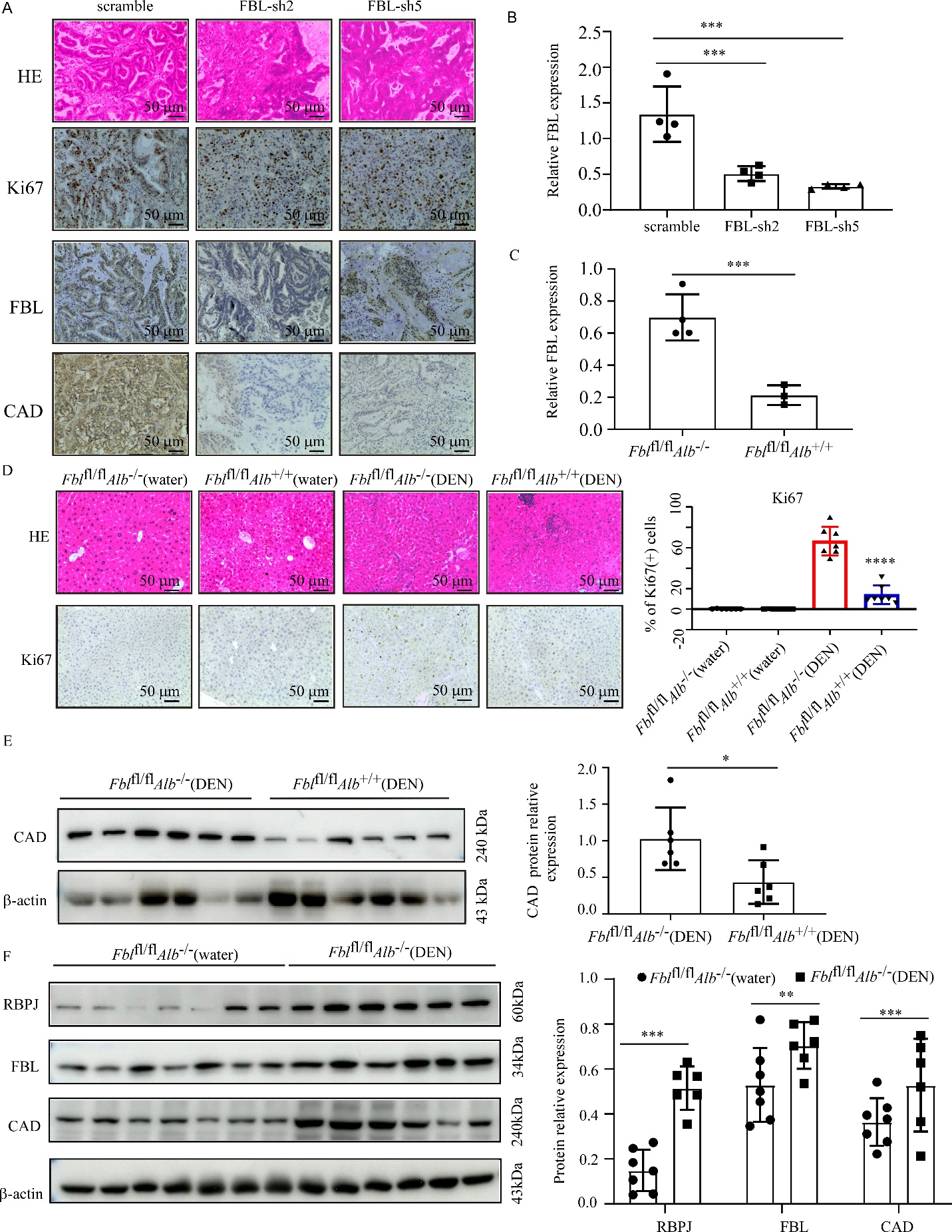
** **Supplementary Fig. 9: The photograph of HE and IHC in the different groups.** A: Graph showing the analysis of the IHC staining of HE, Ki67, FBL, and CAD from PDX tumors. B: Quantitative analysis of the Western blot data illustrated in figure 6E.C: Quantitative analysis of the Western blot data illustrated in figure 6G. D: Graphs showing the quantification of HE, Ki67 staining from different groups mice. E: Western blotting revealed the expression levels of CAD in the *Fbl*^fl/fl^*Alb*^-/-^ (DEN) and *Fbl*^fl/fl^*Alb*^+/+^ (DEN); left: Quantitative analysis of the Western blot data. F: Western blotting analysis of FBL, CAD , and RBPJ protein levels in the *Fbl*^fl/fl^*Alb*^-/-^ (water) and *Fbl*^fl/fl^*Alb*^-/-^(DEN) group; left: Quantitative analysis of the Western blot data. Error bars represent mean ± SD. Significance is indicated by *, *P* < 0.05; **, *P* < 0.01; ***, *P* < 0.001.

**
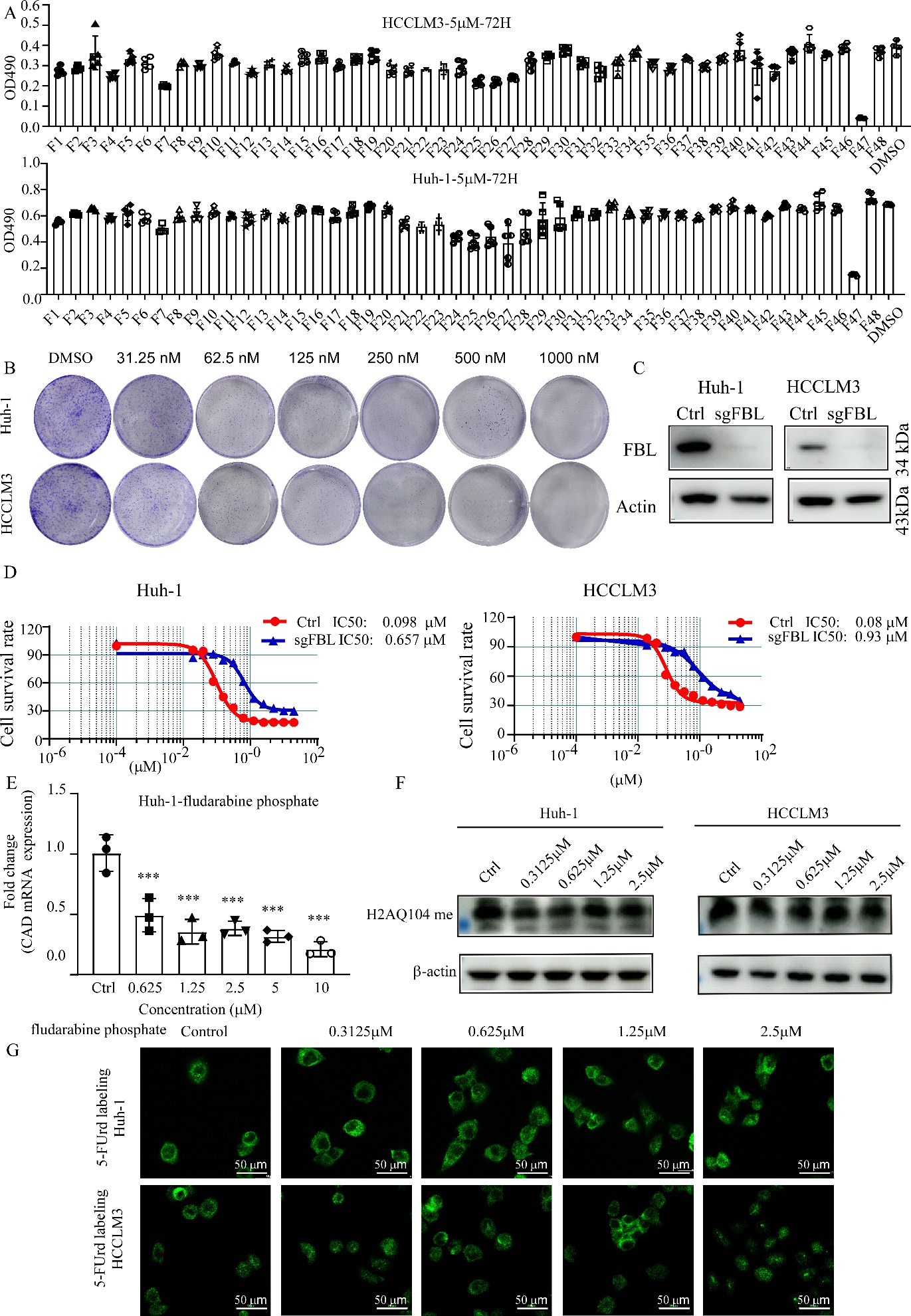
Supplementary Fig. 10: Screen the FBL inhibitors**. A: The MTT of HCCLM3 and Huh-1 cells were treated with 5μM FBL candidate inhibitors. B: The plate colony of Huh-1 and HCCLM3 cells were treated with different concentration of fludarabine phosphate. C: Western blotting revealed the expression levels of FBL in the FBL knockout HCC cells. D: The cell survival rate in the ctrl/FBL-KO HCC cells treated with fludarabine. E: The RT-qPCR analysis of CAD mRNA expression in cells treated with fludarabine phosphate. F: Western blot analysis to verify the H2AQ104me level in cells treated with different concentrations of fludarabine phosphate. HCC cells were treated with different concentrations of fludarabine phosphate for 48 hours, followed by cell harvesting and Western blot analysis. G: Immunostaining of 5-FUrd-labeled RNA in cells treated with different concentrations of fludarabine phosphate. Labeled RNA is stained with anti-BrdU(green). HCC cells were treated with different concentrations of fludarabine phosphate for 48 hours, followed by cell harvesting and immunofluorescence (IF) assay. Error bars represent mean ± SD. Significance is indicated by *, *P* < 0.05; **, *P* < 0.01; ***, *P* < 0.001.

**
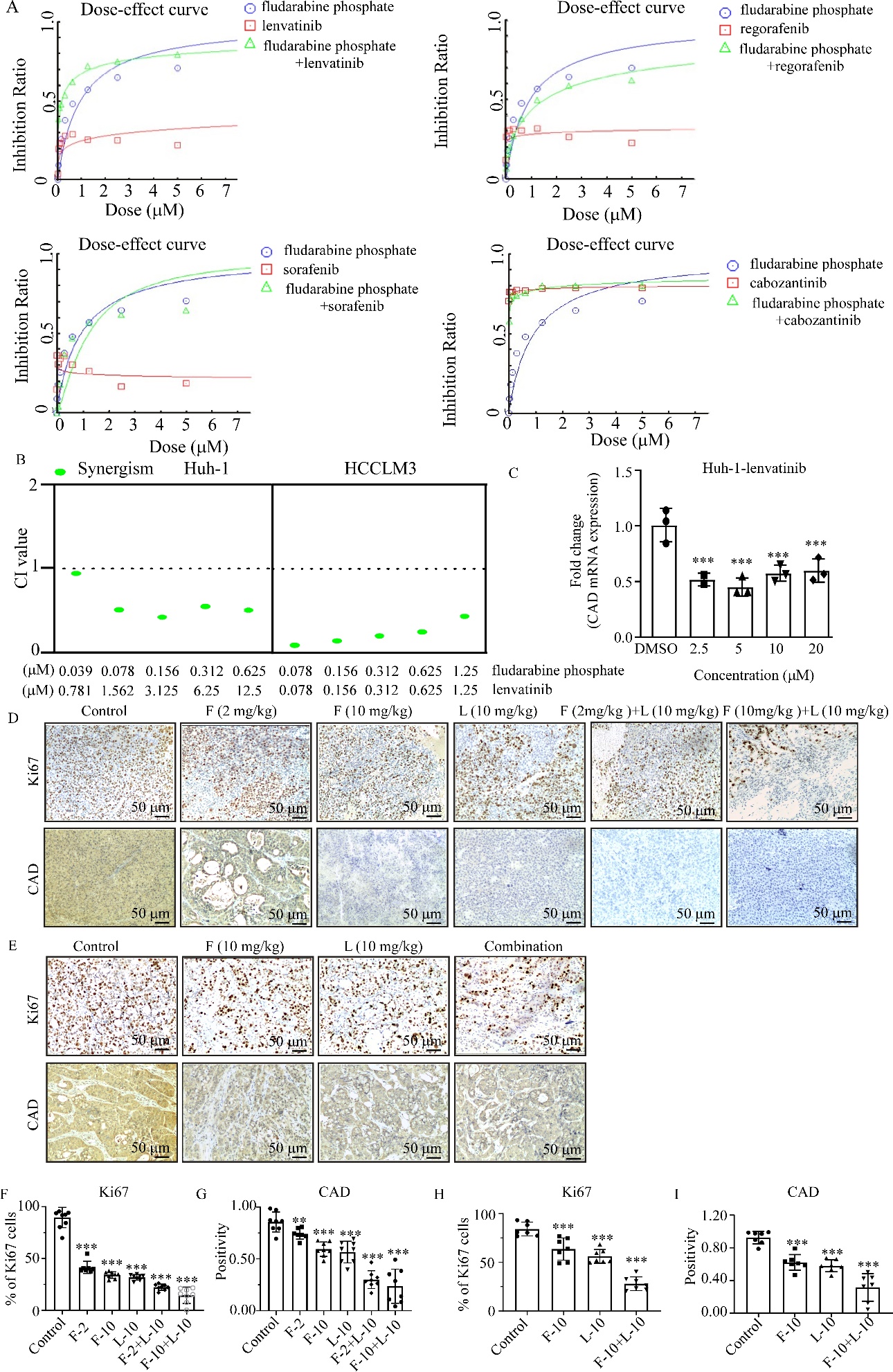
Supplementary Fig. 11: The combination of fludarabine phosphate and lenvatinib drugs.** A: The dose-effect curve of HCCLM3 cells treated with fludarabine phosphate and different drugs. B: Synergism effect of fludarabine and lenvatinib in HCC cells. The synergism and antagonism (CI value) were determined and analyzed using CompuSyn 1.0. CI value > 1.1 indicates antagonism, 1.1 ≥ CI value > 0.9 shows addictive effect and CI value ≤ 0.9 indicates synergism. C: The RT-qPCR analysis of CAD mRNA expression in cells treated with lenvatinib. D: Graph showing the analysis of the IHC staining of Ki67 and CAD from CDX tumors and PDX tumors in the different groups. F, G: The Quantitative analysis of the Ki67 and CAD expression in D. H-I: The Quantitative analysis of the Ki67 and CAD expression in E. Error bars represent mean ± SD. Significance is indicated by ***, *P* < 0.001.

**Supplementary Table S1:**

| **Number** | **Molecular mass** | **Molecular formula** |
| --- | --- | --- |
| F01 | 389.39 | C_16_H_15_N_5_O_5_S |
| F02 | 259.74 | C_12_H_17_NO_3_ |
| F03 | 301.34 | C_10_H_11_N_3_O_4_S_2_ |
| F04 | 402.27 | C_17_H_12_BrN_3_O_2_S |
| F05 | 168.22 | C_6_H_8_N_4_S |
| F06 | 347.36 | C_13_H_13_N_7_O_3_S |
| F07 | 259.7 | C_9_H_13_N_5_O_2_ |
| F08 | 325.4 | C_15_H_15_N_7_S |
| F09 | 285.78 | C_13_H_19_N_3_O_2_ |
| F10 | 189.22 | C_10_H_11_N_3_O |
| F11 | 382.45 | C_18_H_18_N_6_O_2_S |
| F12 | 285.28 | C_14_H_12_FN_5_O |
| F13 | 318.18 | C_14_H_12_BrN_3_O |
| F14 | 286.68 | C10H_11_ClN_4_O_4_ |
| F15 | 179.18 | C_7_H_9_N5O |
| F16 | 286.77 | C_11_H_19_N_6_O |
| F17 | 310.27 | C_14_H_10_N_6_O_3_ |
| F18 | 325.33 | C_16_H_15_N_5_O_3_ |
| F19 | 323.31 | C_13_H_17_N_5_O_5_ |
| F20 | 283.29 | C_13_H_13_N_7_O |
| F21 | 363.81 | C_16_H_18_ClN_5_O_3_ |
| F22 | 377.41 | C_20_H_19_N_5_O_3_ |
| F23 | 288.26 | C_13_H_12_N_4_O_4_ |
| F24 | 379.4 | C_15_H_17_N_5_O_5_S |
| F25 | 283.25 | C_10_H_13_N_5_O_5_ |
| F26 | 386.39 | C_14_H_18_N_4_O_7_S |
| F27 | 242.24 | C_12_H_10_N_4_O_2_ |
| F28 | 349.37 | C_14_H_15_N_5_O_4_S |
| F29 | 268.23 | C_10_H_12_N_4_O_5_ |
| F30 | 280.33 | C_13_H_20_N_4_O_3_ |
| F31 | 256.26 | C_11_H_16_N_2_O_5_ |
| F32 | 261.33 | C_14_H_19_N_3_O_2_ |
| F33 | 185.18 | C_7_H_11_N_3_O_3_ |
| F34 | 419.42 | C_17_H_17_N_5_O_6_S |
| F35 | 341.76 | C_17_H_12_ClN_3_O_3_ |
| F36 | 352.4 | C_19_H_20_N_4_O_3_ |
| F37 | 286.72 | C_14_H_11_ClN_4_O |
| F38 | 366.47 | C_2_1H_26_N_4_O_2_ |
| F39 | 392.39 | C_17_H_16_N_2_O_7_S |
| F40 | 299.8 | C_14_H_21_N_3_O_2_ |
| F41 | 339.44 | C_19_H_25_N_5_O |
| F42 | 299.35 | C_15_H_13_N_3_O_2_S |
| F43 | 388.4 | C_17_H_16_N_4_O_5_S |
| F44 | 254.25 | C_12_H_10_N_6_O |
| F45 | 339.36 | C_17_H_17_N_5_O_3_ |
| F46 | 347.22 | C_10_H_14_N_5_O_7_P |
| F47 | 365.21 | C_10_H_13_FN_5_O_7_P |
| F48 | 285.26 | C_10_H_15_N_5_O_5_ |


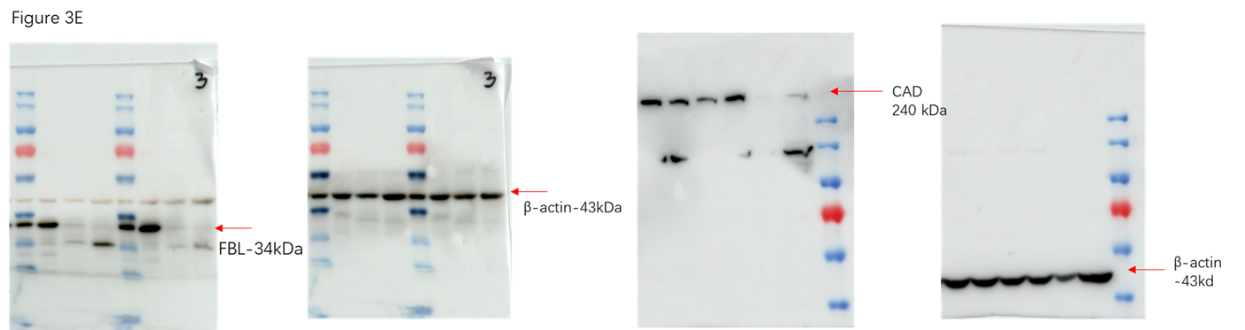

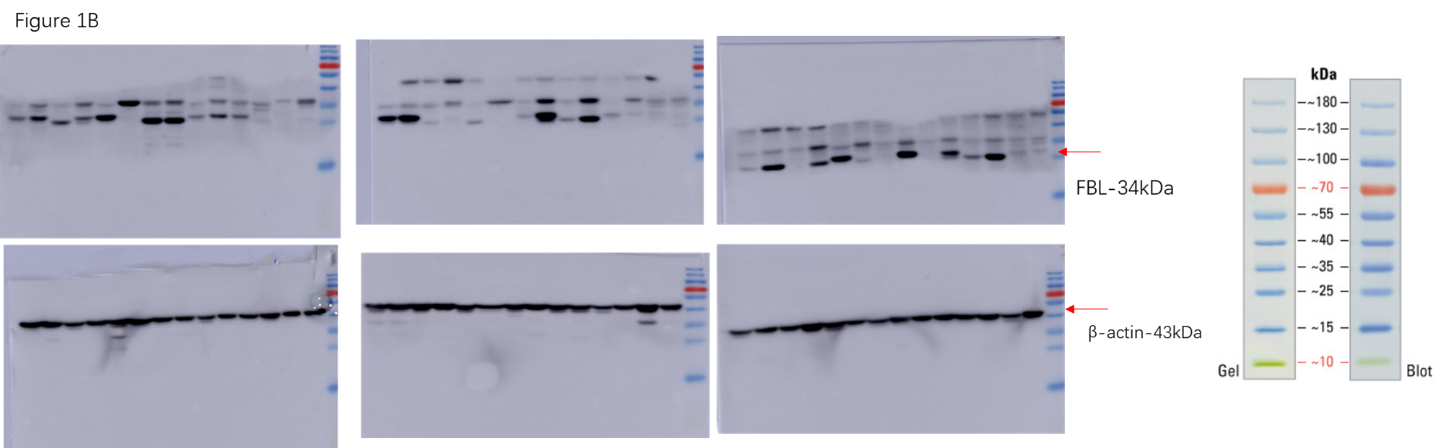

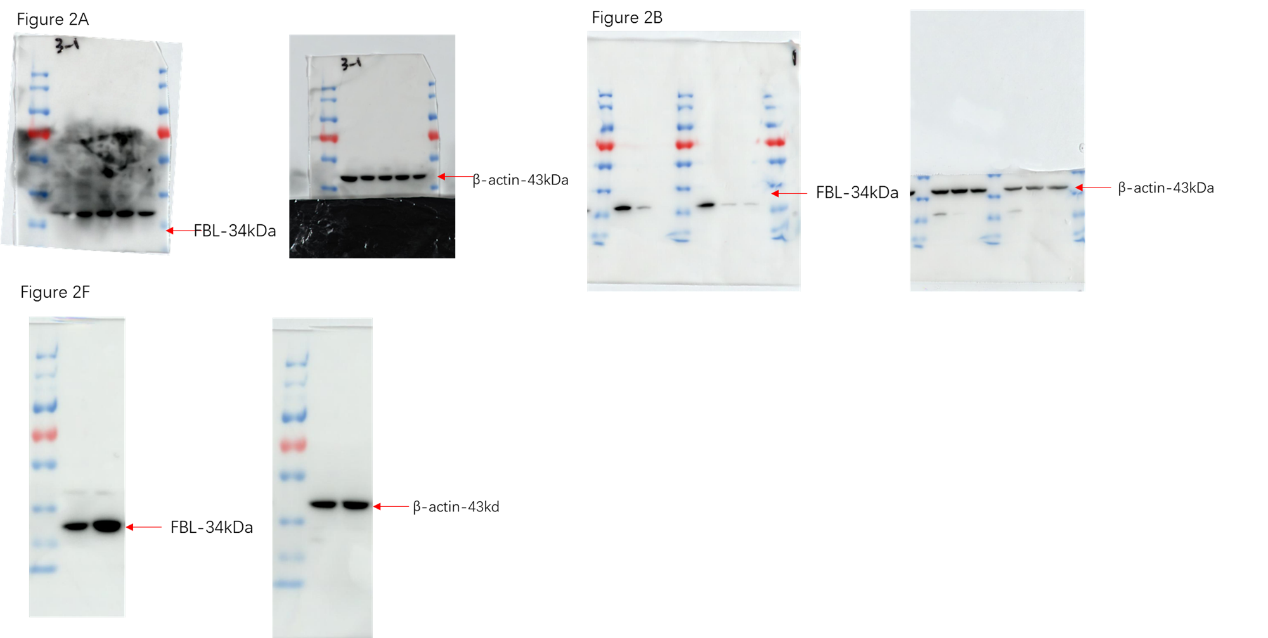


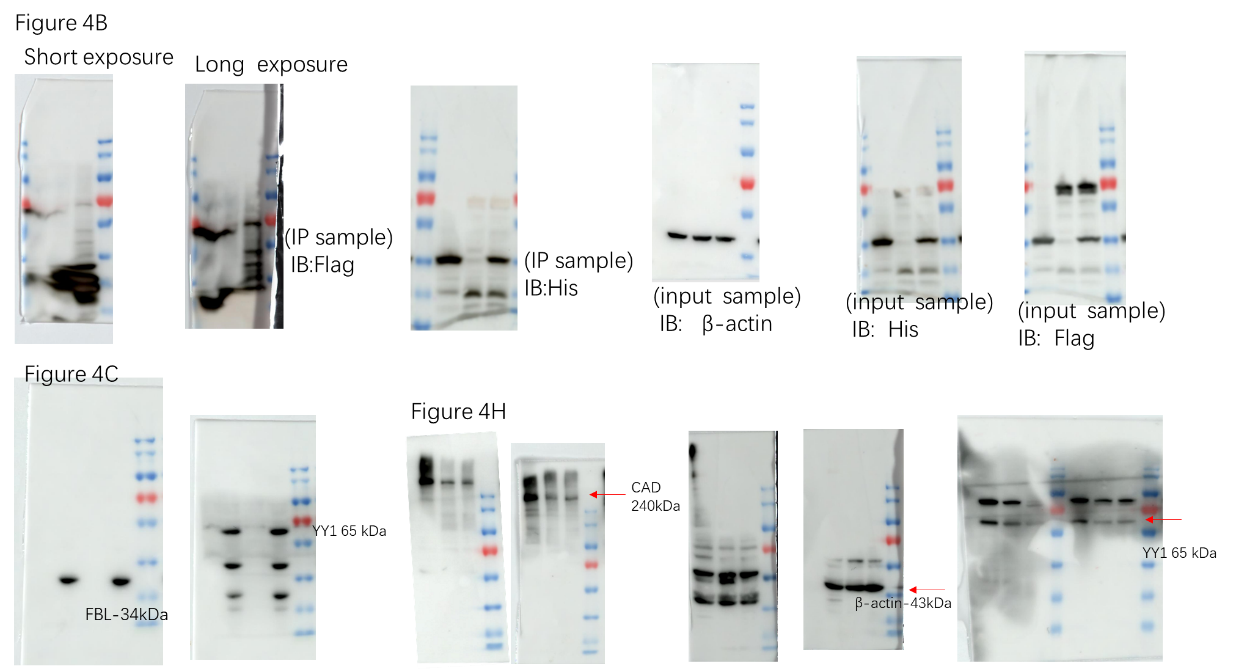


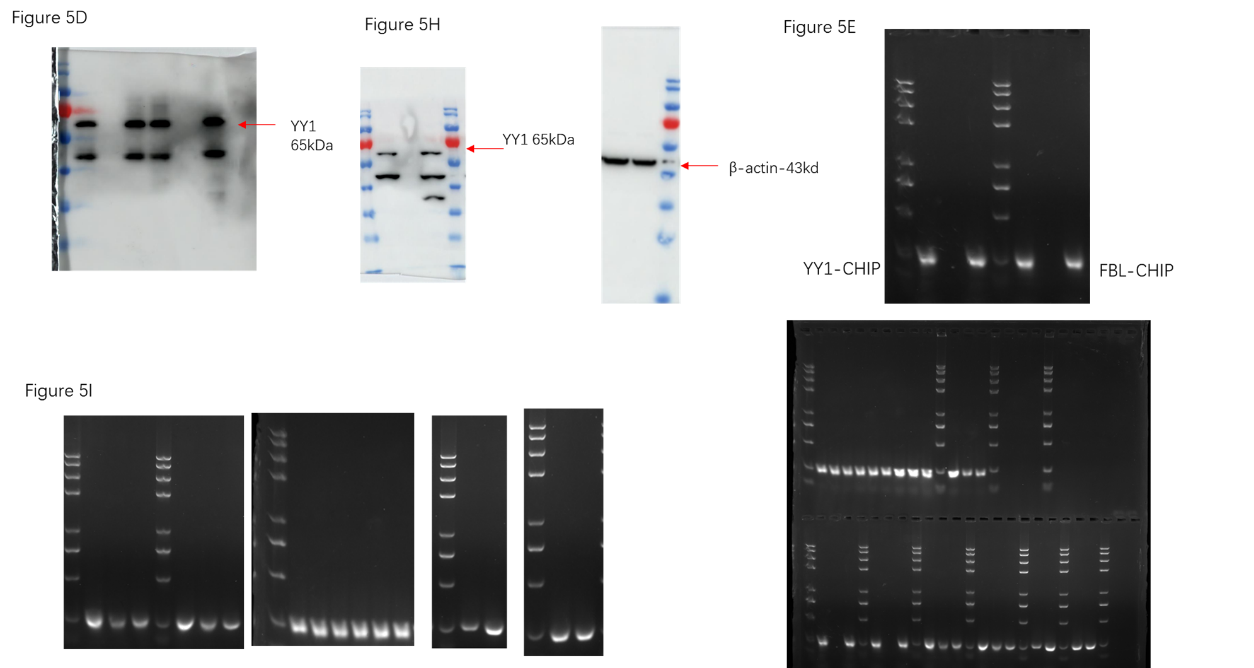


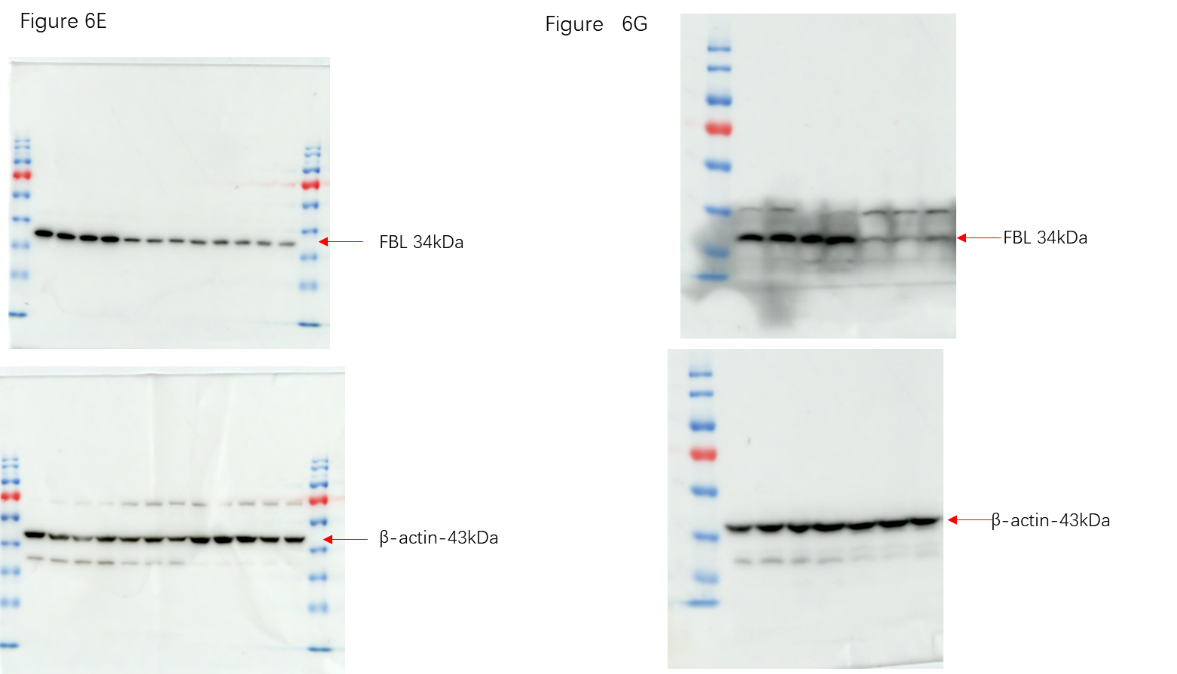

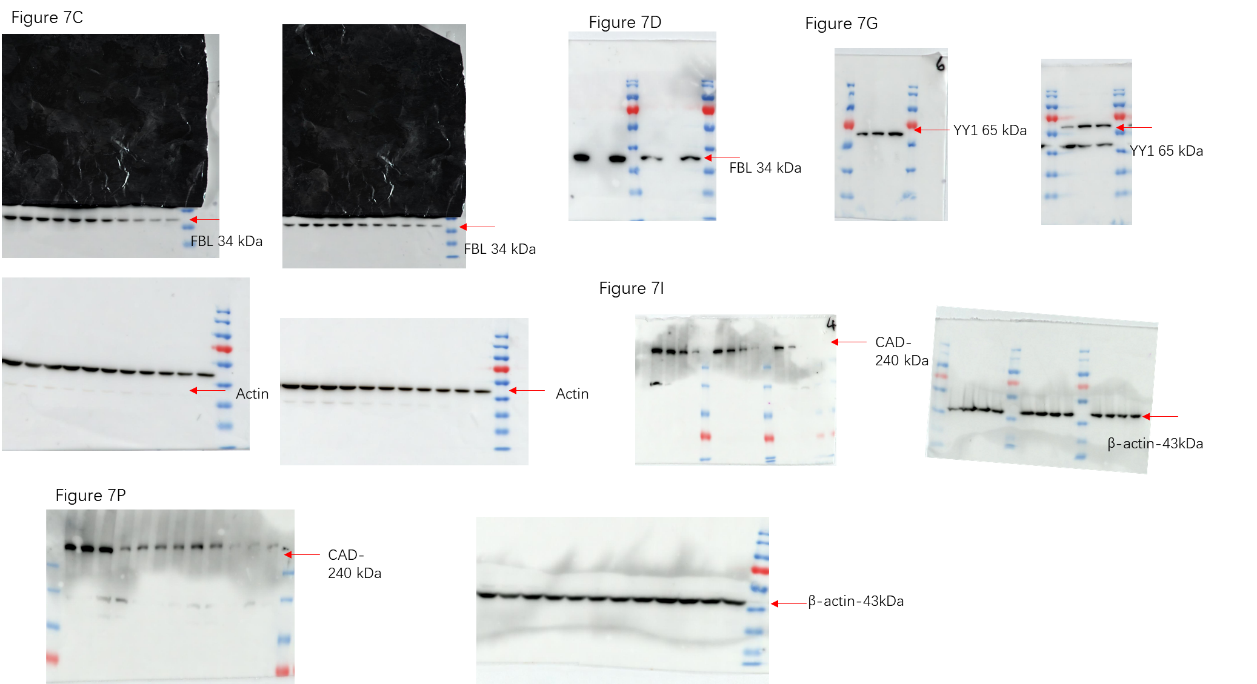


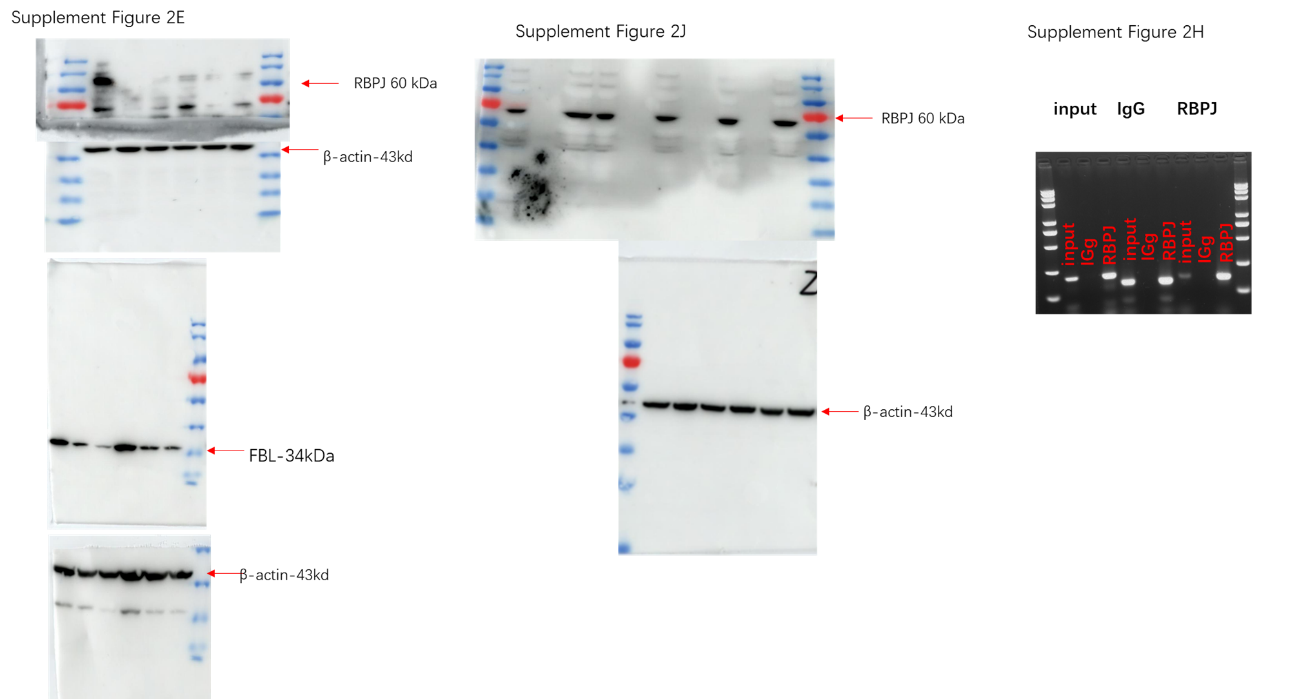


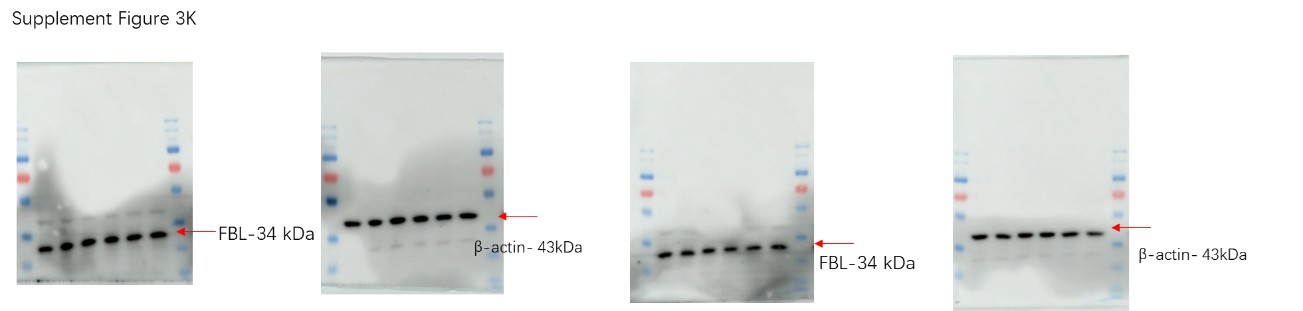

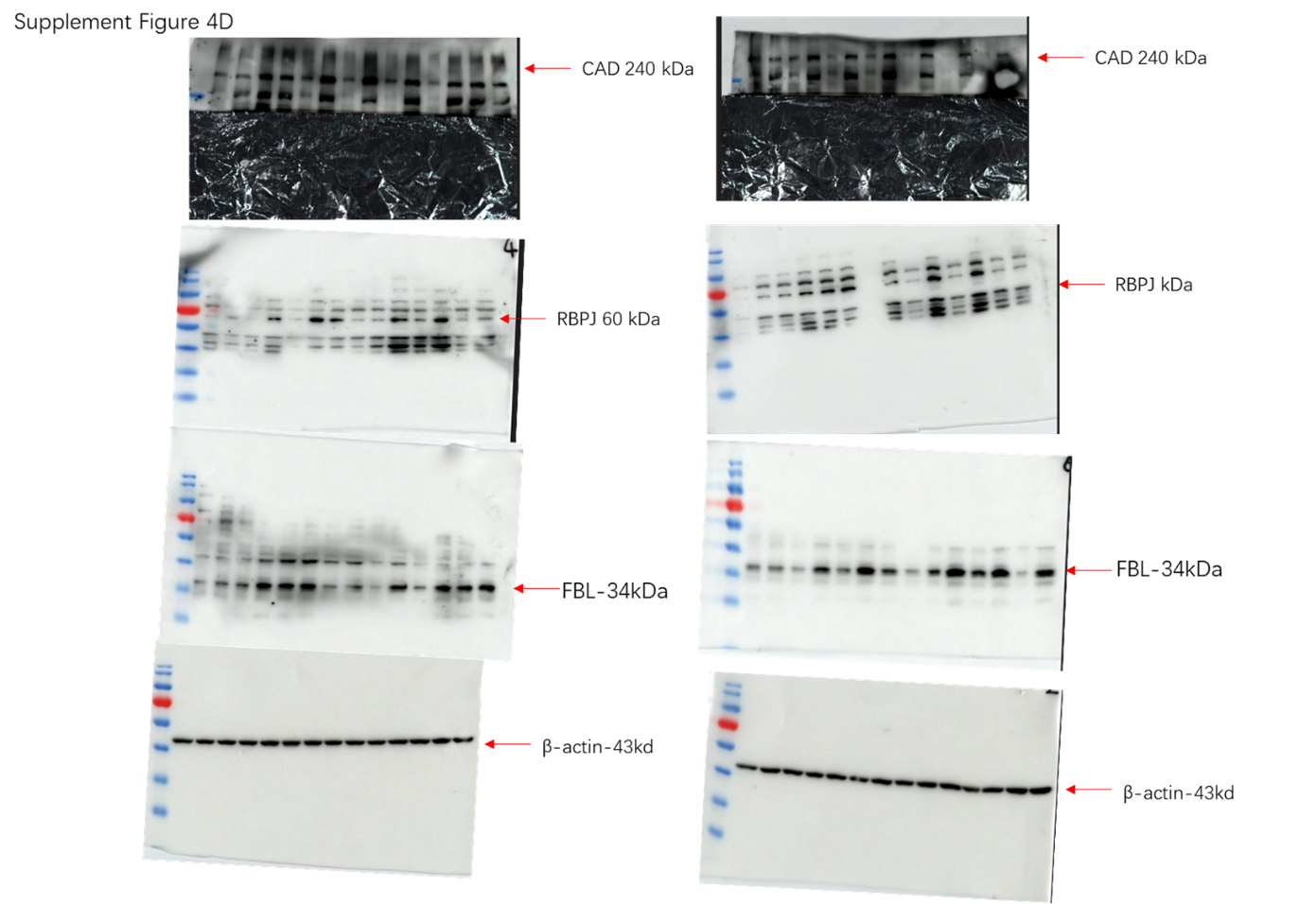

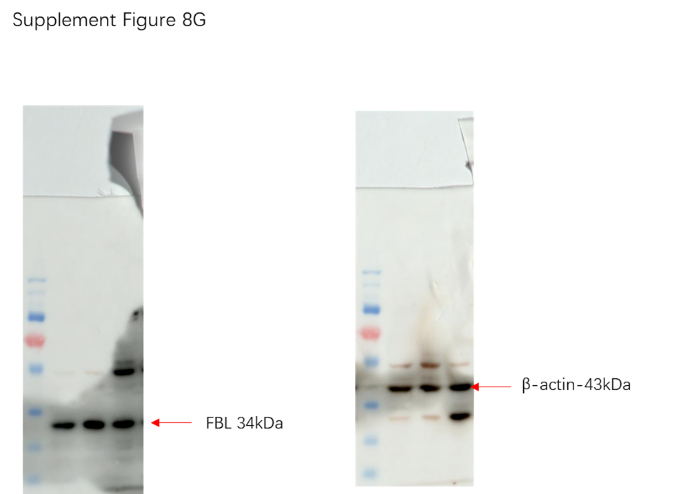


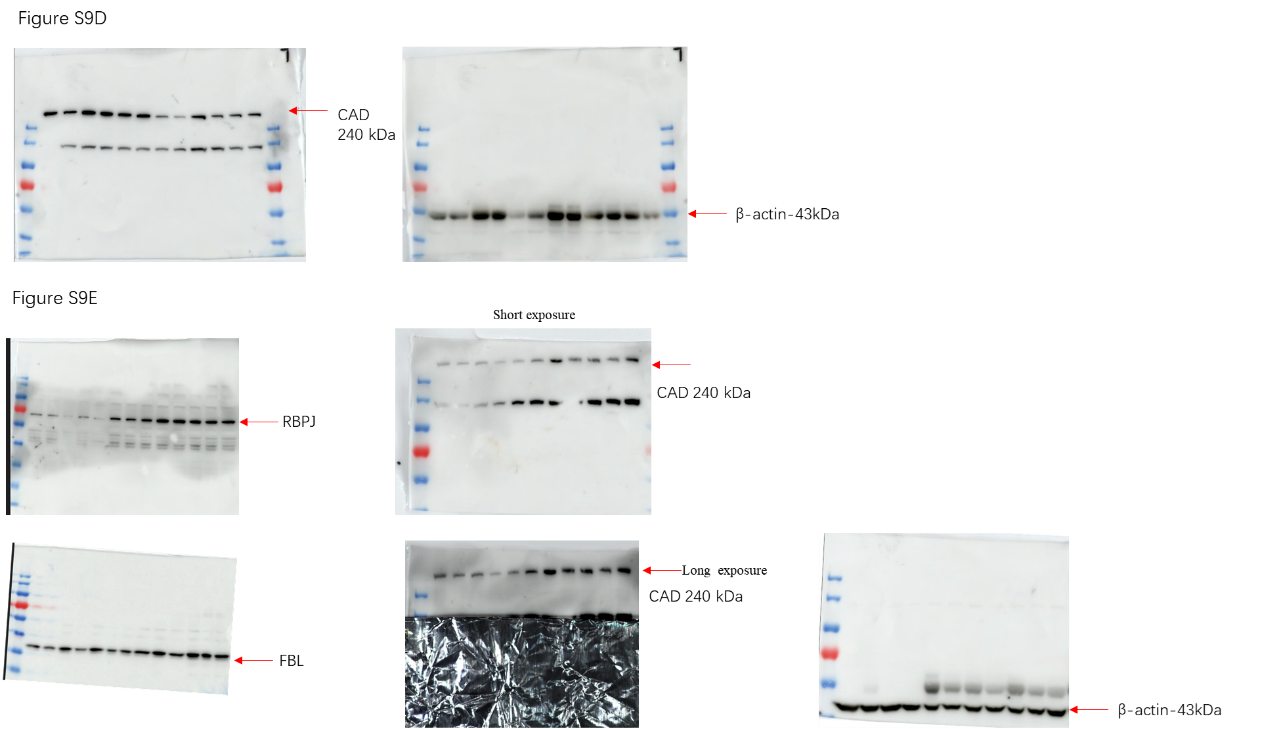


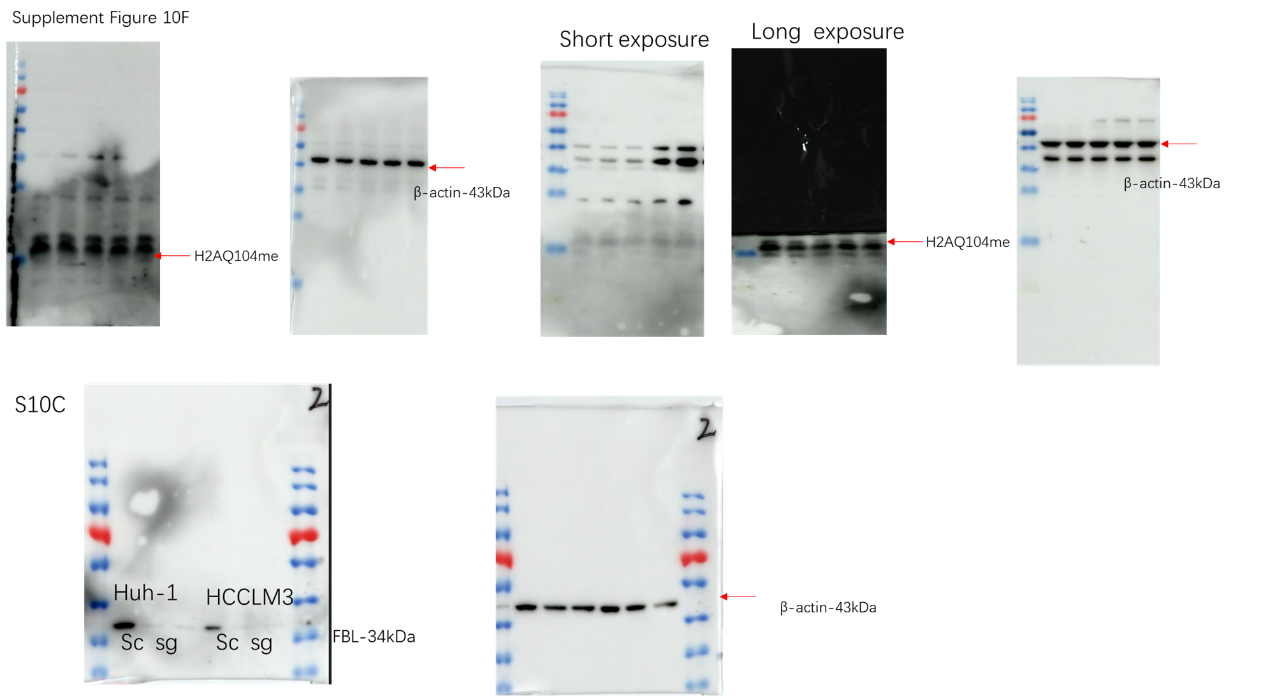

Supplement: Supplementary file 1 — Supplemental Material [file 41419_2025_7684_MOESM1_ESM.docx]
